# Mapping and Characterizing Selected Canopy Tree Species at the Angkor World Heritage Site in Cambodia Using Aerial Data

**DOI:** 10.1371/journal.pone.0121558

**Published:** 2015-04-22

**Authors:** Minerva Singh, Damian Evans, Boun Suy Tan, Chan Samean Nin

**Affiliations:** 1 Department of Plant Sciences, University of Cambridge, Cambridge, United Kingdom; 2 Departments of Asian Studies and Archaeology, University of Sydney, Sydney, Australia; 3 Angkor International Research and Documentation Center, Siem Reap, Cambodia; 4 Department of Forestry Management, Cultural Landscape and Environment, APSARA National Authority, Siem Reap, Cambodia; INRA, FRANCE

## Abstract

At present, there is very limited information on the ecology, distribution, and structure of Cambodia’s tree species to warrant suitable conservation measures. The aim of this study was to assess various methods of analysis of aerial imagery for characterization of the forest mensuration variables (i.e., tree height and crown width) of selected tree species found in the forested region around the temples of Angkor Thom, Cambodia. Object-based image analysis (OBIA) was used (using multiresolution segmentation) to delineate individual tree crowns from very-high-resolution (VHR) aerial imagery and light detection and ranging (LiDAR) data. Crown width and tree height values that were extracted using multiresolution segmentation showed a high level of congruence with field-measured values of the trees (Spearman’s rho 0.782 and 0.589, respectively). Individual tree crowns that were delineated from aerial imagery using multiresolution segmentation had a high level of segmentation accuracy (69.22%), whereas tree crowns delineated using watershed segmentation underestimated the field-measured tree crown widths. Both spectral angle mapper (SAM) and maximum likelihood (ML) classifications were applied to the aerial imagery for mapping of selected tree species. The latter was found to be more suitable for tree species classification. Individual tree species were identified with high accuracy. Inclusion of textural information further improved species identification, albeit marginally. Our findings suggest that VHR aerial imagery, in conjunction with OBIA-based segmentation methods (such as multiresolution segmentation) and supervised classification techniques are useful for tree species mapping and for studies of the forest mensuration variables.

## Introduction

The concept of sacred or culturally important sites protecting small biodiversity-rich forest tracts exists in many countries. As religious and cultural beliefs play an important role in shaping policy and decision making, it has been argued that inclusion of cultural and faith-based beliefs into the conservation paradigm may result in positive outcomes for biodiversity conservation and in protection of endangered species [1,2]. The temple forests surrounding the great monuments of Angkor in Northwestern Cambodia are examples of forested areas that are deeply linked to identity, culture, and sacred beliefs. These areas have evolved along a distinctly different historical trajectory compared to other forests in the surrounding areas because of regulatory frameworks that have defined the former as areas of cultural significance [3–6]. However, in recent decades, Cambodia has seen a sharp increase in deforestation. Culturally important forests such as those in Angkor are facing increased pressure, mainly due to increased infrastructure development and an influx of tourists [5]. Utilization of advanced forest monitoring techniques is crucial for ensuring long-term survival of Cambodia’s forests, sacred or otherwise.

Very-high-resolution (VHR) aerial imagery has increasingly become widely available in recent years. VHR aerial imagery is expected to yield significant benefits for conservation management by facilitating improvement of monitoring of encroachment in protected areas, development of high resolution maps, and species surveys, among other applications [7]. Coupled with image analysis techniques, VHR aerial imagery has been applied to tree canopy research for tree crown measurements and for mapping of tree species of tropical forests in the Brazilian Amazon [8–10] and across the Barro Colorado Island in Panama [11]. These studies have shown that tree crown measurements derived from aerial images correspond closely to field measurements while offering the possibility of answering broader ecological questions pertaining to above-ground biomass (AGB) modeling among other questions.

Image segmentation is an important component of utilization of aerial imagery for forestry studies. Segmentation is intended to identify and isolate individual homogenous objects in an image [12], in this case, tree crowns from VHR aerial imagery. A large number of studies that were focused on individual tree identification and delineation have involved conventional segmentation techniques for isolation of individual tree crowns from aerial data. These techniques may be categorized into two broad categories: (*i*) region growing and (*ii*) boundary detection paradigms.

Watershed segmentation, in particular, is an important member of the boundary detection-based segmentation family [13]. The basic assumption of this method is that treetops contain radiometric maximums that are close to the geometric centers of the treetops [14]. Watershed segmentation has been extensively used for individual tree crown delineation in temperate forests. For instance, Ke and Quackenbush [15] successfully applied watershed segmentation to a maple stand and achieved classification accuracy up to 40%. This algorithm was implemented using VHR aerial imagery acquired over Japanese temperate forests to facilitate the delineation of individual tree crowns and further tree species classification with high accuracy [16]. A modified watershed segmentation technique was implemented by Yang et al. [17] using VHR aerial imagery collected over deciduous woodland in Ontario, Canada. Although the authors discovered that tree crown delineation was strongly linked to tree crown sizes, their algorithm was unable to approximate tree crown shapes well. Research by Jing et al. [14] indicated that the implementation of conventional segmentation techniques such as watershed segmentation is easier in forest ecosystems dominated by coniferous trees as opposed to those dominated by deciduous tree crowns, owing to the relatively more complex structure of the latter.

Delineation of individual tree crowns in tropical forest ecosystems is extremely challenging [18]. Watershed segmentation has been successfully used for identifying oil palm tree crowns using WorldView-2 [19] and for tree crown delineation in North Borneo using IKONOS [20] aerial imagery. However, the basic premise of watershed segmentation limits its use to subtropical and tropical forests, where the determination of radiometric maximums and geometric centers is not straightforward [21,22]. Furthermore, the implementation of conventional techniques such as watershed segmentation is difficult with VHR data owing to high spatial resolution of the latter because the response of individual pixels does not map onto a single entity on the ground [23].

The object-based image analysis (OBIA) paradigm has been specifically adapted for segmentation of individual objects/entities such as tree crowns in VHR imagery [24]. It revolves around partitioning of VHR imagery into non-overlapping objects or segments [15,21], offering the advantage of creating objects that approximate the shape and size of tree crowns in the VHR imagery [25]. OBIA-based approaches may be implemented in conjunction with different algorithms including watershed segmentation [22]. In the present study, the OBIA paradigm was implemented using eCognition software by applying multiresolution segmentation, which is the most commonly used approach. Multiresolution segmentation involves a combination of spectral and spatial heterogeneity of tree crowns for hierarchical region merging [12,26]. Based on these heterogeneity criteria, the algorithm starts by building one-pixel objects. Adjacent individual pixel objects are merged with each other to form meaningful objects based on the heterogeneity criteria [27].

OBIA-based methods have a wide range of applications, ranging from landscape-based analysis to individual tree crown delineation. A significant proportion of the OBIA research on aerial data from the tropics has revolved around landscape level applications. These range from land cover classification to monitoring of invasive species using a combination of Quickbird and Hyperion data [28]. In this research, we briefly focused on examining the application of OBIA-based approaches to VHR aerial imagery for facilitation of individual tree studies. OBIA-based approaches have been implemented using VHR aerial imagery to map disease-infected individual tree species in California [29] as well as to map tree species in Brazil [30,31] and Arizona [32]. Only a handful of research groups have utilized OBIA for crown segmentation in tropical ecosystems as opposed to its application for delineation of individual tree crowns in temperate forests. Tsendbazar [23] has used OBIA (implemented using multiresolution segmentation) for delineating individual tree crowns in high-resolution aerial images and for modeling biomass in the subalpine hill forests of Nepal. In another example, OBIA with multiresolution segmentation was applied to Quickbird data collected over a tropical, eucalyptus-dominated savanna woodland in Australia, and the delineated tree crowns showed a strong overlap with their corresponding reference polygons [33]. Other OBIA-based analyses have been used in conjunction with VHR aerial imagery to distinguish palm trees from surrounding vegetation in the Amazon [34] and for studying tree crown attributes of different species in a tropical urban ecosystem in Brazil [35]. It should be noted that two of these studies have been conducted on a single species within tropical forest ecosystems [33,34] and one in a subalpine forest dominated by coniferous species [23]. Based on existing literature, it can be argued that most methods of OBIA-based segmentation of individual tree crowns in tropical ecosystems have been restricted to systems dominated by a single species, whereas the tree crown segmentation capabilities of OBIA in mixed-species tropical systems have not yet been examined in detail. Further review of literature reveals that OBIA has never been used for detection of individual tree species and for classification within the forests of Southeast Asia [21–23,33].

The efficiency of a given method at isolating and segmenting individual tree crowns varies with the characteristics of the forest itself. It is possible that a segmentation algorithm may work for a given forest type (sparse forests, for instance) but may not work well for a different forest type, such as denser forest stands [36]. Furthermore, most of the segmentation algorithms have been designed for coniferous and temperate deciduous forest stands. These forests have relatively simple tree crown structure compared to tropical forests. The ability of OBIA to carry out accurate tree crown delineation in temperate forests has been established well. However, the effectiveness of OBIA-based segmentation algorithms in tropical ecosystems, especially in mixed-species forests located in the Asian tropics that have complex structures and canopy height reaching 60 *m* [37] needs closer examination. The lack of segmentation techniques specifically designed for tropical forest tree crowns, along with a poor understanding of how existing segmentation techniques work with aerial imagery collected over tropical forests represents a significant gap in the existing methodological toolkit. To the best of our knowledge, only Palace et al. [8] attempted to develop an automated image segmentation technique for high-resolution aerial imagery acquired over a tropical forest.

Further challenges in segmenting of individual tree crowns and in species identification in tropical forests are that a single species may exhibit variable physical parameters, and that two species may have low spectral separation [38]. In other studies, textural features have been included along with spectral information to improve aerial imagery-based tree species classification for temperate forests. Combined bands of spectral and textural information derived from VHR aerial imagery improve the species detection and classification of common temperate trees species such as spruce, pines, and hardwoods by 33% compared to spectral information alone [39]. Combined spectral and textural bands also yielded better classification accuracy than do textural bands alone. A combination of shape, grey-level co-occurrence matrix (GLCM)-derived information, and spectral information has been derived from high-resolution aerial imagery to distinguish and classify commonly found tree species in Sweden [3]. A combination of LiDAR and texture variables has also been successfully employed for commercial tree species monitoring [40,41]. Although GLCM-derived texture features have been effective at improving tree species classification in temperate forests, they have been restricted mostly to biomass mapping [42,43] and land use classification in the tropics [31,44,45]. To the best of our knowledge, texture-based measures have not been previously utilized for tree species detection and classification in the Asian tropics.

Over the past few years, there has been a sharp increase in forest loss in the tropics [46]. Along with the disturbing rates of forest loss in tropical Asia, there have been growing adoption and dissemination of high-resolution aerial imagery and LiDAR data from the tropical forests. Thus, it is important to examine techniques and algorithms that could facilitate the identification of tree species and quantification of forest structure in tropical forests. The development of new algorithms is beyond the scope of this paper. However, by examining the utility of existing approaches for segmenting individual tree crowns and tree species mapping, we hope to show that our results will serve as a benchmark for studies of the structure and species composition of similar forests in the region. The mapping of tropical tree species and evaluation of their biophysical parameters using either aerial or LiDAR imagery have so far been mainly performed on the forests of Neotropics. Here, we present a novel application of these technologies to mapping and measuring the distribution and structure of tree species in Cambodia, with potential applications across Southeast Asia.

## Objectives

Cambodia is home to one of the largest remaining tracts of tropical forests in Southeast Asia [41]. Its forests host valuable timber species and provide habitat to endangered species including the Indochinese tiger. From 2002 to 2006, however, Cambodia lost its forests at the rate of 0.5% per annum, and between 2006 and 2010, its total forest cover declined from 59% to 57%. Although Cambodia still has a significant forest cover, it has been classified as “high forest cover, high deforestation” by the UN-REDD initiative because of the high deforestation rates [47]. Illegal harvesting of trees for luxury timber has taken a heavy toll not only on the forests of Cambodia but also on the surrounding countries. In this region, illegal removal of valuable tree species routinely occurs even within protected areas and wildlife sanctuaries [48].

The present research deals with the mapping of selected tree species in Angkor Thom and the subsequent characterization of their forest mensuration variables. This study is expected to facilitate the monitoring of trees within the tropical forests of Cambodia on a landscape scale. We are using field-measured data alongside VHR aerial imagery and LiDAR imagery, and the primary objectives of this study are (*i*) to evaluate the ability of aerial data to predict the variation in field-measured forest mensuration variables such as tree height and tree crown width and (*ii*) to map individual tree species of selected canopy tree species using VHR aerial imagery. In conjunction with these two objectives, this study also aims (*iii*) to compare the performance of two segmentation approaches- OBIA (implemented using multiresolution segmentation) and watershed segmentation and to determine which of these approaches can predict field-measured tree crown diameters more accurately; (*iv*) to examine which of two classification approaches (maximum likelihood [ML] and spectral angle mapper [SAM]) is best suited for tree species mapping using VHR aerial imagery; and (*v*) to determine how the inclusion of texture features improves classification accuracy.

## Materials and Methods

### Ethics statement

This study was approved by the APSARA National Authority (Authority for the Protection and Management of Angkor and the Region of Siem Reap), who provided permits for the fieldwork described here. No extractive or destructive sampling of tree species was carried out. Only tape measures and clinometer-based measurements of the trees were taken. The following sections describe the study area and measurements in detail.

### The overall approach

The research described here was undertaken with two primary objectives in mind. The first is to evaluate the ability of aerial data to predict the variation in two field-measured forest mensuration variables: (1) tree height and (2) tree crown width. For this purpose, segmentation analysis was applied to the remotely sensed data, and the values obtained from segmentation were compared with their field-measured counterparts. The second objective was to perform the mapping of five individual tree species in the study area using aerial imagery alone. In addition to the spectral bands, texture variables that were derived from these spectral bands were also employed for classification purposes. It must be noted that segmentation approaches were exclusively used to fulfill the first objective (and its associated aims), whereas the classification approaches were exclusively used for fulfilling the second objective. LiDAR data were exclusively used for one purpose only, that is, evaluation of the variability of field-measured tree height. Both objectives (*i*) and (*ii*) involve individual tree data collected in the field.

### The study area

**Fig 1:**
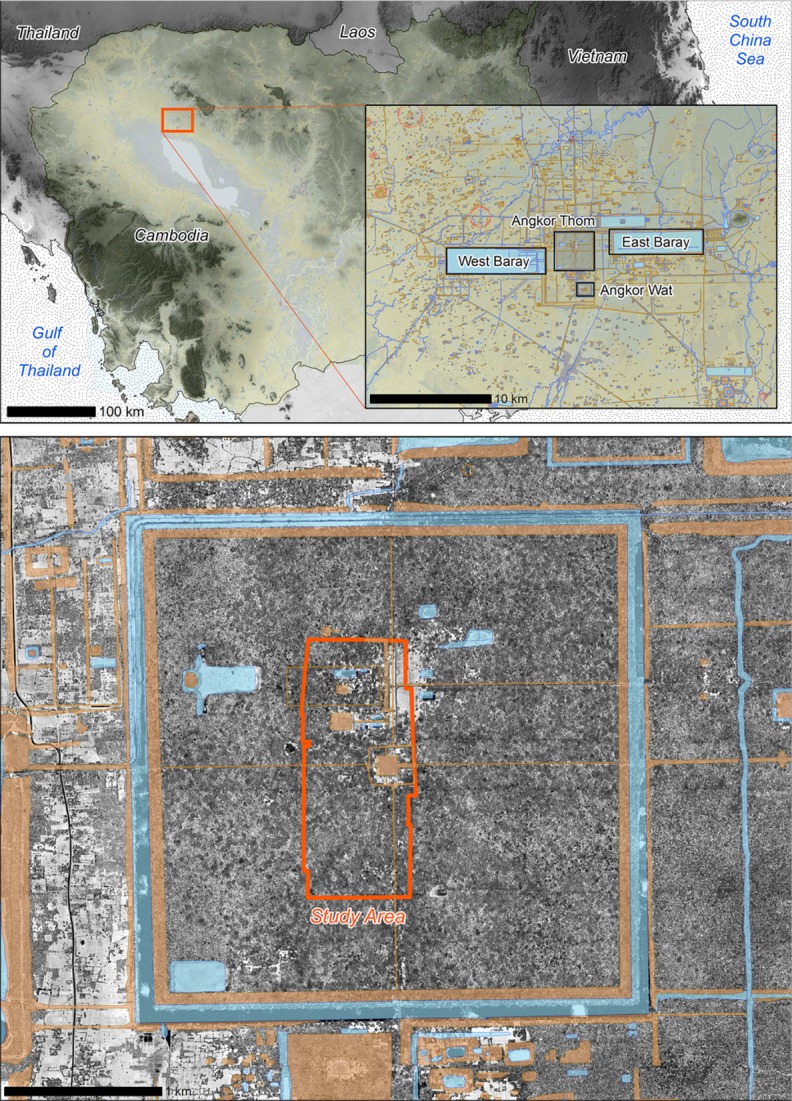
An Overview of the Study Area. Top: A map of Cambodia, shaded relief courtesy NASA/SRTM. Top Inset: An archaeological map of central Angkor, courtesy of the Greater Angkor Project (GAP). Bottom: The study area within the walled city of Angkor Thom, shown against a background of LiDAR intensity data, courtesy of the Khmer Archaeology LiDAR Consortium (KALC), and archaeological data, courtesy of GAP. All remote sensing data for the study were provided by Damian Evans.

Our research was carried out in the heavily vegetated precinct of Angkor Thom, at the center of the Angkor Archaeological Park [49,50]. Fig 1 shows Angkor Thom and our data collection area (within the orange boundary in the lower panel).

The 9-*km* ^2^ area of Angkor Thom includes dozens of significant archaeological sites, most notably the Bayon temple with its iconic face towers. The area also lies entirely within Zone 1 of the UNESCO World Heritage site, which means that this location is under a relatively high level of protection [4]. One can therefore observe a large number of critically endangered tree species (e.g., *Dipterocarpus alatus*) in the area, particularly in close proximity to the monuments and access roadways, although there is evidence of extensive tree felling in areas further away from public view. The vegetation cover in Angkor Thom, which has been variously described as “temple forests or subhumid semi-deciduous forests” or “fragmented evergreen” has undergone cycles of degradation and regeneration during various periods in recent history [4].

### Field data collection

**Fig 2:**
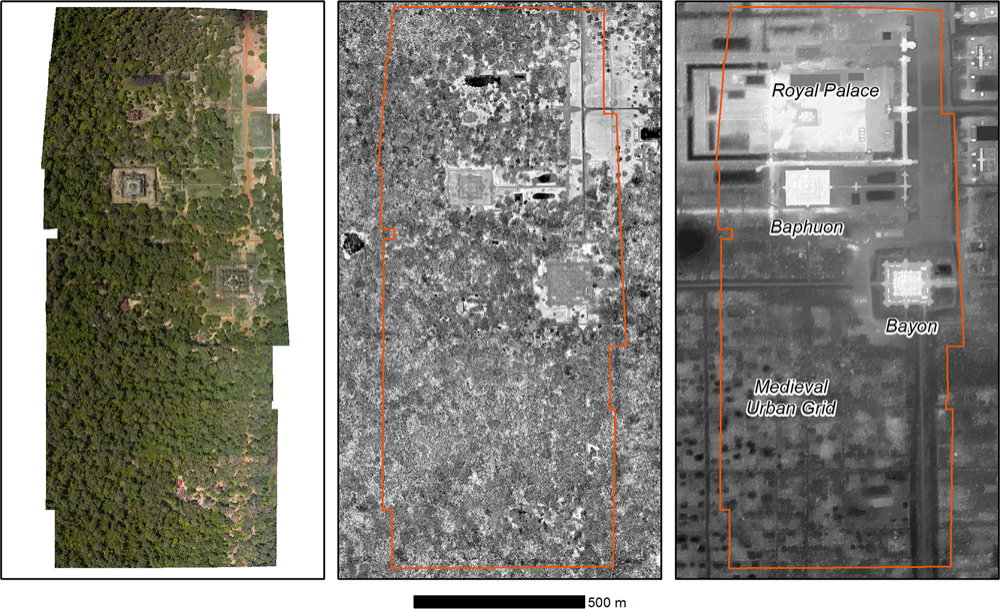
A Detailed View of the Study Area. Left: An orthophoto mosaic. Center: LiDAR intensity data with the study area delineated in orange. Right: A combined digital terrain model and hillshade derived from ground returns in the 2012 LiDAR data, showing temples and features of archaeological interest (all data courtesy of KALC). All remote sensing data for the study were provided by Damian Evans.

The data collection was conducted in December 2013. The forests around Angkor Thom consist mostly of evergreen trees and relatively few deciduous trees. These forests are dominated by tree species whose height typically ranges from 25 to 40 meters. Common tree families include *Meliaceae*, *Dipterocarpaceae*, and *Annonaceae* [51,52]. During the field data collection, the leaves were fully flushed. Because these forests consist primarily of evergreen trees, the issue of deciduous tree flushing can be ignored in this work. Field data collection was performed mostly within forest patches in the immediate vicinity of the Bayon and Baphuon temples, as shown in Fig 2.

Tree height, the diameter at breast height (DBH), and crown width or diameter were measured for trees, both in the immediate vicinity of the monuments and in forest patches located within a 1-*km* radius of the monuments. DBH was measured 1.3 *m* above the ground in accordance with the RAINFOR protocol [53]. Crown width was determined as the average of two perpendicular crown radii, which were measured from the tree bole to tree crown edge using a meter tape [54,55]. The trees were selected using size-stratified random sampling, which ensured that both large trees and relatively smaller trees were sampled from each of the canopy tree species under study.

This survey was focused on canopy trees, which included *D*. *alatus* (57 trees), *Tetrameles nudiflora* (24 trees), and *Lagerstroemia calyculata* (32 trees) among others. The numbers of some of these canopy trees, notably *D*. *alatus* and *H*. *odorata*, are diminishing rapidly in their natural habitat and are therefore on the “List of Threatened Species” of the International Union for Conservation of Nature. These tree species are of great economic importance because they are valuable sources of timber and have been used in forest restoration programs in Southeast Asia [56].

The geographical locations of individual trees were recorded so that they could be located in the high-resolution aerial images. The study area has many prominent architectural structures, most of which have been rigorously surveyed and geolocated using a differential global positioning system (GPS) [57], thereby making them suitable reference points. Trees were geolocated within the imagery using known distances and directions from these reference structures, similar to the strategy employed by Gougeon [58] and others elsewhere, including tropical forest environments [10].

### Aerial data processing

#### Aerial imagery processing

The VHR aerial images were acquired over the Angkor Archaeological Park on April 2013. The imagery consisted of three spectral bands (red, green, and blue) and was captured using a 40-megapixel Leica RCD105 medium-format camera from 800 *m* above the ground. The resulting data have VHR (0.08 *m*) and the individual tree crowns are visually distinguishable. Data preprocessing steps such as radiometric, atmospheric, and geometric correction were carried out by the data provider according to factory specifications. Other parameters such as color balancing and saturation were applied by the data provider based on the light conditions on a given day. All of these preprocessing steps were carried out using the manufacturer’s proprietary software and details of algorithms cannot be revealed. These preprocessing techniques were undertaken with the goal of reducing the impact of atmospheric disturbances, haze, noise, and orientation-related errors [59].

#### LiDAR data processing

The LiDAR data were collected during the same flights as with the aerial imagery, but this time, using a Leica ALS60 laser system installed within an external pod that was mounted on the left skid of a Eurocopter AS350 B2 helicopter. The instrumentation included a Honeywell CUS6 inertial measurement unit, which registered aircraft orientation at 200 *Hz*. Absolute positional information was acquired using a Novatel L1/L2 GPS antenna attached to the tail rotor assembly; the antenna was logging positions at 2 *Hz*. Flying height of 800 *m* above the ground level and the speed of 80 *km*/*h* were chosen to achieve optimal point density, assuming a field of view of 45° for the laser scanner and a default field of 46° for the camera equipped with a 60-*mm* lens. The ALS60 was set to the pulse rate of 120 *kHz* with full waveform acquired across a swath width averaging 650 *m*. In the heavily forested areas of Angkor Thom, which include our study area, the aircraft flew adjacent flight lines in opposing directions with a significant side lap between swaths, and also flew perpendicular flight lines to maximize canopy penetration. Processing of waveform data into discrete points resulted in point clouds averaging ~12 points per *m* ^2^ in the study area. The density of LiDAR point clouds used for forestry applications can vary considerably from study to study, for example, from 1 point per *m* ^2^ [10] to 164 points per *m* ^2^ [60]. Clearly, the latter is very high point density, and such parameters are not commonly available for the tropics. However, it has been shown in various studies that 5-points-per-*m* ^2^ point density and above is adequate for tropical-forestry applications [61,62] and thus, we consider our data to be well within the acceptable range.

An important LiDAR product required for estimating forest parameters (such as above-ground biomass [AGB], tree height, and forest structure) is the canopy height model (CHM). A CHM is a three-dimensional (3D) surface that characterizes the height of vegetation across a landscape [54].

Vegetation height data or the CHM is obtained by subtracting a digital terrain model (DTM) from a digital surface model (DSM). A DTM is 3D representation of the elevation of the ground surface without vegetation cover [63]. In contrast, a DSM includes the ground elevation and all objects above it (such as trees). The DSM is obtained from the first LiDAR data return, whereas the DTM is obtained from the final returns, which may often represent the ground surface [54]. Because the final return might also originate from an object above the ground, smoothing and filtering algorithms are needed to obtain an estimate of ground surface elevation [64]. In this study, the CHM and DTM were both extracted using the FUSION software with the Ground Filter algorithm [65] (adapted from Kraus and Pfeifer [66]). It uses a linear prediction algorithm for each measurement as an iterative process. Three iterations of the algorithm are sufficient to extract the probable ground points from the 3D point cloud. To obtain the continuous DTM, the discrete 3D point cloud was projected onto a 2D grid in the X-Y plane. The grid was divided into grid cells of equal dimensions along the X- and Y-axes. A mean elevation of all the points falling onto the same grid cell was assumed for each grid cell. The result was a raster image representation of the 3D point cloud, with pixels corresponding to the grid cells and pixel values as the mean elevation for the corresponding grid cells.

The raster obtained from coarse ground points that was returned by the Ground Filter algorithm is expected to have abrupt changes of elevation within a neighborhood of grid cells, which is not a good representation of the actual surface, as ground elevation does not change abruptly. To prevent these problems, a Median Filter was applied using a window of 3 × 3 grid cells. By translating a window of 3 × 3 grid cells across the whole grid, the value of each grid cell was replaced with the median of values of all the grid cells in that window. This approach should result in uniform elevation values throughout the raster and thus produce a better representation of the actual ground surface. A detailed workflow of how the CHM was produced from LiDAR point cloud data is presented in the S9 Fig.

### Predicting field-measured forest mensuration variables from aerial data

**Fig 3:**
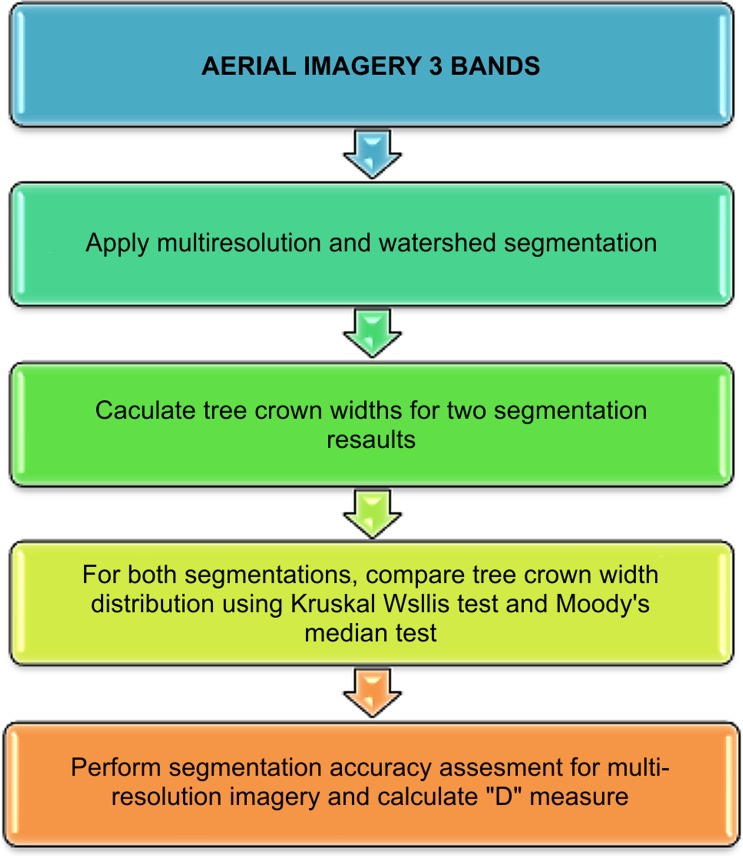
Extraction of Crown Width Values from Aerial Imagery.

Multiresolution segmentation and watershed segmentation algorithms were applied to aerial imagery to extract crown width values, as depicted in Fig 3. These extracted values were then compared with field-measured values to determine which of the algorithms could predict ground-measured values more accurately.

**Fig 4:**
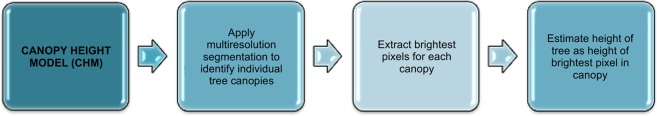
Extraction of Tree Height Values from LiDAR-derived CHM.

Multiresolution segmentation was also applied to the LiDAR-derived CHM to extract tree height data, as illustrated in Fig 4. LiDAR-derived tree heights were then statistically compared with field-measured tree heights. From the CHM, multiresolution segmentation was used to identify individual tree canopies. The brightest pixels of each canopy were extracted to facilitate the estimation of tree height.

#### Image segmentation

Segmentation is a process via which pixels in one or more images are grouped into segments/objects that share a homogenous spectral similarity and make sense in the real world, in this case tree crowns [24]. The watershed segmentation technique used in this study works by converting image data into a gradient scale image that allows the image to be viewed as a grayscale topographic surface. On the topographic surface, the darkest grey values represent low points and the brightest ones represent the high points of the surface. The low points of the grayscale act as valleys. Starting from these minimum values of the image, the surface is filled with water until water spills over to the watershed of the adjacent valley. Once this water-filling is completed, the entire area is separated out into contour basins. These basins are the delineated tree crowns [67]. Further details of this method can be found in the study of Chen et al. [68]. This procedure was implemented using MATLAB.

Using the eCognition software, the OBIA paradigm was applied separately to the aerial imagery and to the LiDAR-derived CHM to extract individual tree crown widths and heights, respectively. The basic assumption of the OBIA paradigm is that a group of pixels in an image can be congregated to form a geographic object. The congregation of individual pixels into homogenous objects is based on the similarity of digital number (DN) values as well as spectral and shape policies [69]. There are several segmentation algorithms that can be used to operationalize the OBIA paradigm. The multiresolution segmentation algorithm used in this study employs a bottom-up approach wherein a one-pixel object is created. This one-pixel object is expanded to mimic an actual object on the ground by joining it with adjacent objects. The parameters that were considered during joining of these pixel-objects to form an actual geographic object (in this case, a delineated tree crown) included spectral and shape heterogeneity criteria and the compactness ratio [70]. Delineation was performed using the procedures specified by Jakubowski et al. [71].

**Fig 5:**
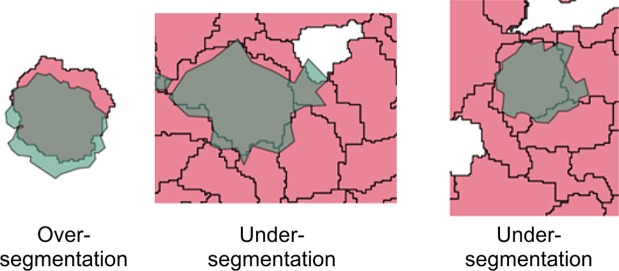
Oversegmentation versus Undersegmentation. The green polygons are the manually digitized polygons that were overlaid on multiresolution segmentation polygons (shown in pink).

A very common approach to checking the accuracy of trees delineated using an automated algorithm is to evaluate the “goodness of fit” of the segmented tree crowns. A typical method of doing so involves using manually digitized polygons as a reference and evaluating the “closeness” of the algorithm of segmented tree crowns [43]. The Closeness Index (*D*) estimates the “goodness of polygon matching” between the reference and the segmented tree crown polygons [38,72]. Twenty-five random tree crowns were manually digitized from the VHR aerial images, as shown in Fig 5. The corresponding aerial imagery-segmented tree crowns were then overlaid on these, and the *D* value was calculated using the following equation:

$$D=\sqrt{{\left(Oversegementation\right)}^{2}+{\left(Undersegementation\right)}^{2}}$$ (1)

This *D* measure accounts for the oversegmentation and undersegmentation that may have occurred during automated delineation. Undersegmentation means that a segment (in this case, a tree crown segmented from aerial imagery) contains a significant crown part of more than one tree. Oversegmentation refers to more than one segment being associated with a ground tree. Fig 5 provides a visual representation of oversegmentation and undersegmentation.

Both of these situations are commonly encountered during tree crown segmentation [73]. In addition, this method of overlaying segmented polygons on manually digitized polygons allows researchers to evaluate the accuracy of spatial location of segmented tree crowns as well as their topology and geometric shapes [43]. The closer the *D* value is to zero, the more accurate is the segmentation [74]. For more details of this method, please see S11 Fig.

#### Statistical analysis

One of the main aims of this research was to determine how well aerial data-derived forest mensuration variables correspond to field-measured values of the same. Prior to any analysis, the adherence of the data to conditions required for most parametric tests was verified. Conditions of normality of residual distribution, assessment of linearity between variables, and examination of association between the independent variables and residuals were evaluated in the R programming language. A QQ plot of the residuals revealed that most of the points fell on the QQ line, except for the tail points. Heavy tails such as those observed in this case are indicative of errors being non-normal (see S2 Table). Given the fact that the data do not strictly adhere to the conditions requisite for parametric tests, nonparametric alternatives have been used. Spearman’s rank correlation was used to quantify the strength of association between values derived from the field and aerial data.

Another aim of this research was to determine which segmentation algorithm (watershed or multiresolution) better predicts the ground-measured crown diameter. Residuals of crown widths measured in the field and those extracted from aerial imagery using multiresolution and watershed segmentation techniques showed deviation from normality. Hence, nonparametric methods were used for the comparison of these. The Kruskal-Wallis test was employed to see whether significant differences existed between the crown widths obtained from different sources. Mood’s median test was used to determine whether multiresolution segmentation-derived crown widths or watershed-derived crown widths are significantly greater or smaller compared to field-measured crown widths.

### Tree species classification

Tree species classification was carried out using either three-band spectral data only or using three-band spectral data in conjunction with texture data. The following section describes the different processes undertaken to carry out tree species classification.

#### Classification quantifiers

**Fig 6:**
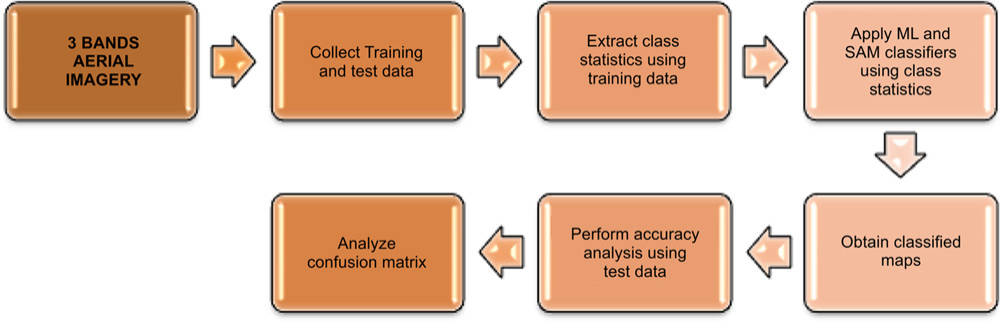
Applying Classifiers to Aerial Imagery.

Two classification algorithms were used for mapping of individual tree species, namely, ML and SAM techniques. Seventy percent of the ground-collected tree location information was used for classification, whereas the other 30% was used for validation. The split between classification and training datasets was performed randomly [43]. Both are supervised classifiers that use statistics obtained from the training data for classification of unclassified data. The ML algorithm calculates the probability of a given pixel belonging to a given class. Each pixel is assigned to a class that has the highest probability. Unless a minimum probability threshold is specified, all pixels are classified [75]. SAM quantifies similarity between training pixels and unclassified pixels by measuring the angle between the class-mean vector obtained from the training data and the vector of unclassified pixels. The pixel is assigned to the class for which the angle is found to be the minimum among all classes, as shown in Fig 6. Essentially, the spectral similarities between the reference and target objects are quantified and used for classification purposes [75].

Both classification techniques were implemented using the ENVI 5.0 image processing software. Several measures of accuracy such as overall accuracy, kappa coefficient, and producer’s and user’s accuracy values have been utilized for validating the classification results [76]. Tree species classification using spectral and texture bands were carried out using both of these supervised classifiers. However, the following two steps were taken to generate the texture bands: texture analysis and feature selection.

### Texture analysis

**Fig 7:**
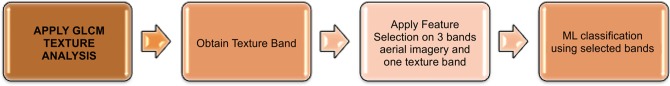
Applying GLCM Texture Analysis to Aerial Imagery.

The concept of GLCM was proposed by Harralick [77] in 1973 and this approach is designed to quantify and describe regions of interests in an image. Although the concept of texture is inherently qualitative, for the purpose of image analysis, texture indicates the spatial variation of pixel values and tonal heterogeneity along a certain direction in the image [42,43]. GLCM measures are usually classified into two categories: first-order and second-order statistics. First-order statistics or occurrence statistics (such as mean, variance, and entropy) do not take into account the relationship among pixels. These statistics are simply designed for quantification of variation in tonal frequency around a given pixel. Second-order statistics or a co-occurrence matrix (such as contrast and angular second moment) is used for quantification of the frequency of association between brightness value pairs [43]. GLCM values were extracted from the three different bands of the VHR aerial imagery using the ENVI image processing software. Fig 7 shows application of GLCM texture analysis.

Further details of the different GLCM texture measures are available in [43,59].

#### Feature selection

Feature selection refers to the process of selecting a subset of features (or in this case, bands) that can best differentiate separate objects [78]. One of the aims of this study was to determine whether inclusion of textural information can improve species classification and differentiation. As a first step, it is important to find out which texture features best distinguish the tree species from one another. Feature selection was focused on selecting a subset from the original data (in this case, texture bands) that best describes the properties of the target objects [79]. This has been done via a rank ordering algorithm (the gain ratio feature selection algorithm). This method attempts to assign weight to each feature or band (in the case of multispectral or hyperspectral imagery) based on how much information is contained in each feature or band. The information content of a feature or band is estimated using the training data available. This is a standard way of ranking features or bands because the more information contained in a feature or band, the more useful it is for classification purposes. The bands with low weight or information content do not contribute much to improving the classification accuracy and are therefore discarded [80]. This algorithm was implemented using both the open-source software WEKA and the FSelector package of the R programming language.

## Results

### Multiresolution and watershed segmentation methods

The Kruskal-Wallis test was performed among the field-measured, multiresolution-derived, and watershed segmentation delineated individual tree crowns. The Kruskal-Wallis test of differences between the distributions of the field-measured crown widths and multiresolution segmentation-based delineated individual tree crown widths yielded a value of 3.13, which meant that there was no statistically significant difference between these two distributions. On the other hand, this test of the difference between field-measured and watershed-segmented aerial tree crowns yielded a value of 84.48, with the significance level 0.001 (indicating that the latter are significantly different from field-measured values). The median of the widths of watershed-segmented tree crowns was 8.7 m, the median of the field-measured crown widths was 17 m, and the median of the multiresolution segmentation segmented tree crowns was 18.18 m. To further evaluate the medians, the Mood test of difference of medians was conducted. The test showed no differences between the medians of field-measured and multiresolution segmentation-based tree crowns. However, it showed a highly significant difference between the medians of the samples of watershed-segmented and field-measured tree crowns. The median of the width of watershed-segmented tree crowns was significantly smaller than the medians of the two other samples. On the basis of both Kruskal-Wallis and Mood’s test of median difference, it can be concluded that multiresolution segmentation is a sound algorithm for individual tree crown delineation, whereas watershed segmentation underestimates field-measured crown tree widths. A histogram of crown width distributions that was obtained with these three methods is presented in S7 Fig.

### Forest mensuration variables from the aerial data

**Fig 8:**
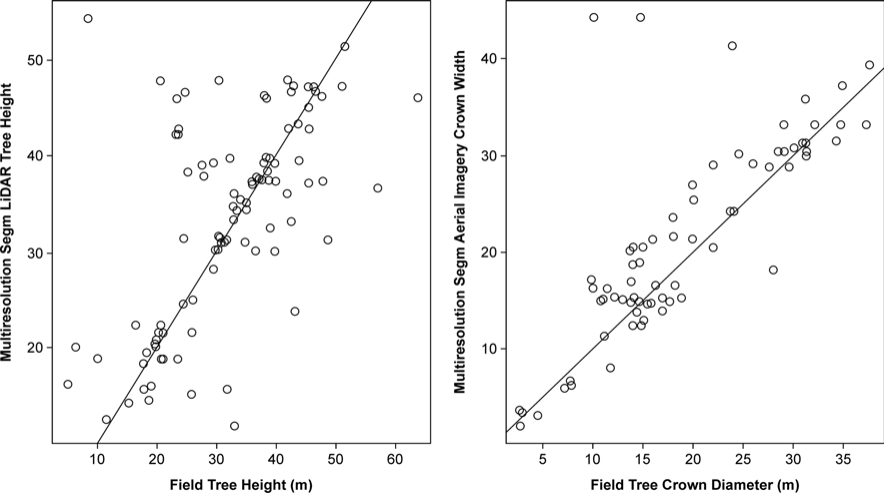
Comparison of Field-measured and Object-based Image Analysis (OBIA)-predicted Crown Widths and Tree Heights.

Very few forest mensuration and tree allometry data have been collected for the forests of continental Southeast Asia. To the best of our knowledge, forest mensuration data have not been collected in the forests of Cambodia. Multiresolution segmentation was performed on aerial imagery to extract the width of tree crowns and on the LiDAR-derived CHM for extracting tree height. Multiresolution segmentation-based values were compared with the field-measured values of tree crown diameters and field tree heights, as shown in Fig 8.

As depicted in Fig 8, the field-measured and multiresolution segmentation-based predicted crown widths and tree heights showed a positive correlation within each category. The Spearman’s rank correlation coefficient (rho) between field-measured and LiDAR-derived tree heights was 0.589, whereas rho between field-measured and aerial imagery-derived crown widths was 0.7825. A 1:1 line (shown in red) was also fitted, and it can be seen that the predicted values for tree crowns from multiresolution segmentation coincide strongly with the field-measured values. The root-mean-square error (RMSE) for field-measured tree heights and LiDAR heights was 1.28 *m*, whereas that for crown widths was 2.23 *m*. The reference aerial data (manually digitized tree crowns) and multiresolution segmentation-based tree crowns showed a *D* value of 0.318 (i.e., accuracy of 69.22%, 31.78% error) indicating that multiresolution segmentation-based tree crowns had been segmented with 69.22% accuracy.

Comparison of field-measured and multiresolution segmentation-measured crown width and tree height values was performed on the three species that are commonly found in the vicinity of the temples: *D*. *alatus (*57 trees), *Tetrameles nudiflora* (24 trees), and *L*. *calyculata* (32 trees). Table 1 provides a summary of the average (± standard error) field-measured and multiresolution segmentation-predicted mensuration variables for the three aforementioned tree species.

**Table 1:** Field-measured and Light Detection and Ranging (LiDAR)-predicted Mensuration Variables for Some Tree Species.

Tree Species	Tree Height (*m*)			Crown Width (*m*)		
	OBIA-Predicted	Field-Measured	R^a^	OBIA-Predicted	Field-Measured	R^a^
***Dipterocarpus alatus***	34.3±1.57	36.5±1.3	0.416	23.53±1.37	20.9±1.2	0.777
***Tetrameles nudiflora***	30.58±2.9	31.81±3.49	0.717	16.08±1.84	15.26±1.91	0.754
***Lagerstroemia calyculata***	31.43±3.89	35.8±4.28	0.743	21.5±4.6	21.15±4.5	0.685

^a^Spearman’s rank coefficient of correlation.

Furthermore, tree height and crown width are known to correlate with tree DBH [74]. The strength of association between field-measured tree height and crown width and field-measured DBH was evaluated using Spearman’s rank correlation, which yielded a coefficient of 0.40 and 0.202, respectively, with ground-measured DBH.

### Classification of tree species

#### Feature selection results

The gain information ratio selection algorithm ranks features in the order of importance. Out of all the texture features, the entropy of the red band was identified as the only feature that had discriminating power between the different classes under study.

#### Spectral and texture bands

**Fig 9:**
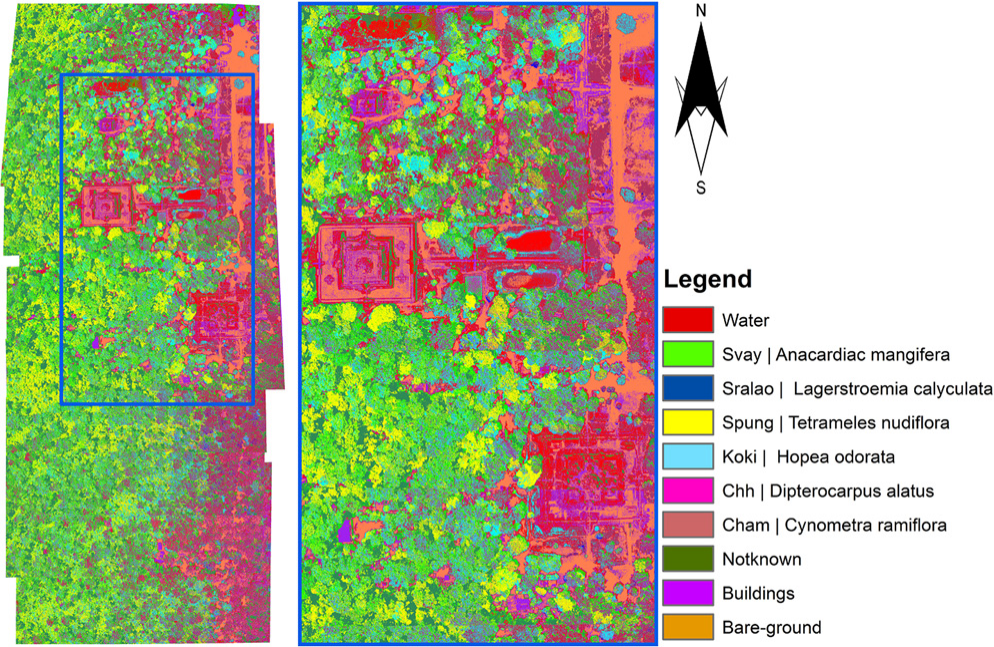
A Classification Map of Tree Species from Three-band Aerial Imagery. CHH: *Dipterocarpus alatus;* KOKI: *Hopea odorata;* SPUNG: *Tetrameles nudiflora;* SRALAO: *Lagerstroemia calyculata;* CHAM: *Cynometra ramiflora*; and SRL: *Lagerstroemia calyculata*.

Classification was carried out using the three-band aerial imagery and a data stack that included spectral bands along with the texture band selected using the gain information ratio algorithm. Out of these, the results from ML classification of three-band aerial imagery showed the highest overall accuracy (83%) and kappa coefficient (0.76). These results were retained as the final classification map of tree species, as shown in Fig 9.

For the ML-derived tree map classification (from the three-band image), the producer’s and user’s accuracy levels varied between 0 and 99.8% and between 0 and 85%, respectively. A detailed summary of producer’s and user’s accuracy values of different classes is presented in Table 2.

**Table 2:** Comparison of Producer’s and User’s Accuracy Levels for Three-band Maximum Likelihood Classification.

	Buildings	CHAM	CHH	KOKI	SPUNG	SVAY	SRL	Water	Unknown	Bare-ground
**Prod. Acc.%**	86.3	52.9	0	87.1	85.6	93.7	0	96.2	88	99.8
**User Acc.%**	25.6	22.5	0	86.2	94.7	34	0	63.2	85.3	76.3

CHAM: *Cynometra ramiflora* CHH: *Dipterocarpus alatus;* KOKI: *Hopea odorata;* SPUNG: *Tetrameles nudiflora;* SVAY: *Anacardiac mangifera* and SRL: *Lagerstroemia calyculata*.

Examination of producer’s and user’s accuracy levels suggested that high confidence can be placed on the detection and mapping of individual classes, except for *D*. *alatus* and *L*. *calyculata*. In these two cases, both producer’s and user’s accuracy levels were zero, suggesting that the ML classification of three-band data was not suitable for resolving these classes. The ML classification was performed on a combination of spectral and texture bands (i.e., the texture band entropy of red band was combined with the existing three spectral bands of the aerial image). Although the overall accuracy and kappa coefficient did not improve, the detection and separability of tree species—notably *D*. *alatus* and *L*. *calyculata*—improved slightly. The former now showed producer’s and user’s accuracy of 8% and 20%, respectively, whereas the latter showed accuracy of 1.8% and 0.7%, respectively. SAM classification with three spectral bands yielded the overall classification accuracy of 35.78% (with kappa coefficient 0.2341) and then 33.85% (with kappa coefficient 0.223) with the texture band (fourth band) included. This value was low compared to that of ML classification, which was 83.27% (with kappa coefficient 0.7510) for three-band data and 73.83% (with kappa coefficient 0.6238) for four-band data.

## Discussion

### Comparing multiresolution and watershed segmentation methods

We show comparison of multiresolution segmentation and watershed segmentation in terms of delineating individual tree crowns from VHR aerial imagery. Multiresolution segmentation approximates the field tree crowns with a high level of accuracy in terms of measurement of crown width, geometry, and spatial location. The field-measured and multiresolution segmentation-extracted crown widths have a strong association with each other (rho = 0.7825).

Watershed-segmented tree crowns, on the other hand, significantly underestimate tree crown width. Review of existing literature indicates that watershed segmentation yields underestimated tree crown widths in other forest ecosystems as well [81]. This is because distinguishing tree crowns exclusively on a radiometric basis can reduce the performance of watershed segmentation [82]. Although watershed segmentation works well for coniferous trees owing to their conical shape, the performance of the algorithm deteriorates in deciduous and other broadleaved forest types [83]. For more complex tree crowns (such as those located in the tropical forests), watershed segmentation has the drawback of oversegmentation and identifying spurious tree tops [14]. Furthermore, watershed segmentation is known to significantly overestimate LiDAR-derived tree height [84]. Research by Huang et al. [85] indicated that implementation of OBIA-based techniques yields better estimates of crown width than does watershed segmentation alone. The *D* value of segmentation accuracy (30.78%) indicates that the multiresolution segmentation-based tree crowns match the corresponding field crowns in terms of spatial location, dimensions, topology, and the geometric shape, with a 69.22% level of accuracy. This closeness index correlates with the analysis of multiresolution segmentation accuracy carried out by Kumar et al. [21], Singh [43], and Mbaabu [74] in different tropical ecosystems. The segmentation accuracy results indicate the utility of multiresolution segmentation-based tree crown segmentation for a mixed-species tropical ecosystem in Southeast Asia.

### Use of aerial data for studying forest structure variables

OBIA-based segmentation was applied to a LiDAR-derived CHM for obtaining estimates of tree height. The LiDAR-derived tree height data have a moderately strong correlation with field-measured heights (rho = 0.589). Predicting tree heights (including individual tree height) is an important part of LiDAR-based studies of forestry and is fraught with significant technical problems. The most common source of uncertainty in LiDAR-derived tree height data stems from deriving DTMs under the forest canopy cover, which in turn translates into errors in estimating the LiDAR tree height [82]. Forest canopy cover and vegetation structure is known to yield up to a 3.67-*m* error in LiDAR tree height estimates in forest ecosystems, notably for tropical forests [86,87]. The presence of a dense understory or interaction with multistoried canopy structure (as is the case in our study area) can further reduce the accuracy of height assessment. Examination of the sources of error in field-measured heights was performed by Larjavaara and Landau [88]. They noted that the clinometer-based approach (used in the present study) essentially produces 1:1 correspondence with actual tree heights, but the height of taller trees is significantly overestimated (sometimes by 100%). This approach produces low systematic error but high random errors. Topographic factors also influence LiDAR-based estimates of tree height. Although these are more pronounced in the presence of steeper slopes, they are not completely absent in flat terrains [86]. Individual LiDAR tropical tree height data can have error ranging from 3% to 20%, where RMSE between field and LiDAR tree heights (for emergent trees) can be up to 7 *m*. Finally, LiDAR is also known to underestimate field tree height in the tropical ecosystems [89]. Research carried out by Imai et al. [90] in the temperate urban forests near Tokyo indicated that prediction accuracy of LiDAR-based tree height data varies across different tree species. All of these factors contribute to the error between field-measured and LiDAR-extracted tree height data.

Furthermore, these factors are significantly magnified in a tropical ecosystem. Tree height measurements with LiDAR in the tropics can have significant sources of uncertainty [81], and this uncertainty and errors in our LiDAR-derived height values are within the range of what has been observed in other tropical forest studies. The RMSE value of 1.28 *m* and strength of association between field- and LiDAR-derived values in this study is consistent with RMSE values between field-measured and LiDAR tree height data from other tropical ecosystems [86,91], and the LiDAR-derived tree height values are within the range of ground tree height values observed in similar ecosystems [51]. Thus, we can conclude that multiresolution segmentation is a suitable algorithm for taking tree height measurements on a landscape scale. Research by Jakubowski et al. [71] indicated that multiresolution segmentation-derived forest mensuration variables are comparable to those produced by the 3D segmentation of point clouds in terms of measurement accuracy. In the absence of 3D segmentation approaches for tropical forests, it may be argued that OBIA-based methods such as multiresolution segmentation are useful for examining tree structures on a landscape scale.

### Tree species classification using spectral and texture bands

This study is the first of its kind to use VHR aerial imagery in conjunction with field-collected geolocation data on tree species to generate a classification map of tree species of a tropical forest ecosystem in Southeast Asia. Here, we utilized both spectral data and a combination of spectral and textural information when carrying out tree species classification of the five tree species. Combining spectral and textural data helped to improve the separability and classification of all tree species under consideration, albeit marginally, in case of tree species that could not be identified on the basis of spectral information alone. This is a potentially important finding. As mentioned previously [38], tropical tree species can have low spectral separability, and this is the case for some of the tree species assessed in the present study (see S5 Fig). One of the species that could not be identified by spectral data alone is *D*. *alatus*. This is an endangered species, whose numbers have declined significantly in the study area. Hence, the spectral response of this species is arguably dominated by that of the surrounding trees. Hyperspectral bands yield higher separability of tree species, but these data are not available for the study area. Thus, we resolved to determine whether texture features could improve species separability. Texture features have not been previously used for identification of individual tree species and for classification in a tropical ecosystem, although their utility for classification of temperate tree species is well documented. Entropy texture information was previously and successfully implemented for tree species classification in Canada by Coburn and Roberts [92].

The ability of texture measures, in particular red band-based ones, to distinguish between different tropical forest types has been discussed by various authors, including Singh et al. [43], Zhou et al. [42], and Eckert [93]. This research builds on the existing findings by demonstrating the utility of texture measures from the landscape level down to individual tree species and by facilitating classification of tree species in tropical ecosystems. Indeed, the separability of the two species that could not be identified by spectral data alone improves marginally with the inclusion of texture data. Given that many species such as *D*. *alatus* are endangered and isolated, the collection of extensive field-based records on these species is not feasible. Thus, increasing the number of spectral bands or using hyperspectral data may yield better results. In the absence of hyperspectral data, incorporating texture measures may improve tree species mapping in other similar tropical ecosystems in the region. Furthermore, this research involves evaluation and comparison of two classification techniques, i.e., ML and SAM, and established the utility of the former in producing an accurate tree species classification map of the study area. Comparison of classification techniques conducted by Cho et al. [94,95] also showed that ML produces the highest classification accuracy in mapping tropical savanna tree species using high-resolution multi-spectral data.

Many previous studies on tropical tree species mapping, notably those by Jansen et al. [96], Garzon-Lopez et al. [9], and González-Orozco et al. [10] involved mapping of tropical tree species in the Neotropics using manual delineation techniques (which require extensive field data collection). Extensive field data collection is not possible in Cambodia owing to the presence of land mines. Thus, the use of supervised classification in conjunction with field tree location data collected from accessible areas can facilitate tree species mapping on a landscape scale, as demonstrated in this study (S6 Fig describes why supervised classification maybe used over manual delineation techniques for tree species mapping). In addition, supervised classification was applied to high-resolution aerial imagery and World View-2 imagery, and high classification accuracy levels for individual species were obtained. To the best of our knowledge, only one other research group has attempted to use supervised classification for tree species identification and classification on aerial imagery in a tropical mangrove forest ecosystem [97]. On the basis of this research and that of Heenkenda et al. [98], it can be argued that aerial imagery in conjunction with appropriate techniques may facilitate landscape scale monitoring of tree species for a few select tree species.

It must be noted that aerial imagery cannot replace hyperspectral imagery for large-scale classification of tropical tree species. VHR aerial imagery has a limited number of spectral bands, whereas hyperspectral data have hundreds of spectral bands and thus, the latter are more suitable for large-scale classification of tree species in tropical forests, as described in several studies. For example, Cho and colleagues successfully identified and mapped eight savannah tree species in the Kruger national park using aerial hyperspectral data [95]. Clarks and Roberts [97] also used hyperspectral data for successful classification of seven tree species to a high level of accuracy in the tropical forests of Costa Rica. Other studies have utilized a combination of hyperspectral data, LiDAR, and conventional aerial imagery for mapping of tree species in temperate forests [99,100]. Given a choice between hyperspectral and aerial imagery, the former is clearly preferable for tree species mapping on a landscape scale in a tropical forest ecosystem. However, hyperspectral data cannot resolve the confusion between tree species in all cases [101]. Furthermore, owing to initiatives such as conservation drones [7], high-resolution aerial imagery is cheaper to acquire compared to LiDAR and hyperspectral data. As demonstrated in this work and in that carried out by Heenkenda et al. [98] and Garzon-Lopez et al. [9], aerial imagery does offer the potential of classifying 3–4 tree species with high accuracy, even though it cannot help with detailed large-scale classification of tree species as hyperspectral data can.

## Conclusion

This research deals with the mapping of tree species and characterization of their forest mensuration variables around the temples of Angkor Thom, Cambodia, within the Angkor Archaeological Park. Using field-measured data in conjunction with VHR aerial imagery and LiDAR data, this study shows that aerial data can readily predict variation in field-measured forest mensuration parameters such as tree height and tree crown width. Additionally, comparison of two segmentation approaches in OBIA—multiresolution segmentation and watershed segmentation—reveals that the former is more effective than the latter at approximating ground tree parameters. Two classification approaches were also examined—ML and SAM—and ML was found to be more accurate at classifying the features. Moreover, an additional band was found to increase the classification accuracy of the two approaches.

This study is the first to successfully apply OBIA to the mapping of tree species within a protected area in Cambodia and to characterization of the forest mensuration variables of those trees. This development has important ramifications for practical biodiversity conservation and monitoring on the ground. The use of such tree species maps provides researchers with crucial information on tree species dynamics in difficult-to-access areas and helps to identify patterns of tree/forest loss at an early stage.

The research described above has not only obvious practical applications in terms of understanding and preserving natural landscapes but also important implications for conservation and management of cultural heritage. In many areas of the world, such as Southeast Asia and Mesoamerica, forests are recognized as fundamental components of the cultural landscape that are otherwise primarily defined by spectacular temple complexes [4,5,102–105]. Archaeological evidence indicates that unsustainable land use contributed to the downfall of “tropical forest civilizations” of antiquity [57,106–109]. Although their monuments were largely reclaimed by the forests over the last millennium, the landscapes of the Maya and the Khmer are once again hot spots of deforestation [5,110]. The sheer size of these cultural landscapes can place a tremendous burden on the resources of the developing nations in which these landscapes are typically located. That burden, however, can be partially alleviated by means of effective application of geospatial technologies [80]. Here, we demonstrated one such application, where aerial data can be used to effectively create an inventory of tree stock within a protected area, which can serve as a foundation for management and conservation of temple forests within protected sites.

## Supporting Information

S1 Fig QQ Plots of Tree Height Residuals- Thick Tail Distributions (Indicative of Non-normal Distribution of Errors). (DOCX)

S2 Fig QQ Plots of Field Crown Width Residuals- Thick Tail Distributions (Indicative of Non-normal Distribution of Errors). (DOCX)

S3 Fig QQ Plots for All Three Species. (DOCX)

S4 Fig Comparison of Field-measured and Object-based Image Analysis (OBIA)-Predicted Crown Widths and Tree Heights. (DOCX)

S5 Fig Spectral Separability of the Different Tree Species Under Consideration. (DOCX)

S6 Fig Maximum Likelihood Classification of Spectral and Textural Bands for Tree Species Classification (4 Band Imagery). (DOCX)

S7 Fig Distribution of Crown Widths- of Field Measured Data, eCognition (Multiresolution) Extracted and Watershed Segmentation Extracted. (DOCX)

S8 Fig LiDAR Point Cloud Processing for Deriving CHM and Related Products. (DOCX)

S9 Fig Derive Tree Heights from LiDAR CHM Using Multiresolution Segmentation. (DOCX)

S10 Fig Segmented Tree Crowns from Aerial Imagery. (DOCX)

S11 Fig Evaluating Segmentation Accuracy Using Goodness-of-Fit. (DOCX)

S1 Table Field Measured DBH, Tree Height and Corresponding LiDAR Height (These Data Were Collected sans Species Information). (DOCX)

S2 Table Data Summary of Field Measured DBH, Tree Height and Corresponding LiDAR Height. (DOCX)

S3 Table Field Measured DBH, Field Measured Crown Width, OBIA Extracted Aerial Imagery Crown Width, Watershed Segmented Aerial Imagery Crown Width (These Data too Were Collected sans Species Information). (DOCX)

S4 Table Data Summary Field Measured DBH, Field Measured Crown Width, OBIA Extracted Aerial Imagery Crown Width, Watershed Segmented Aerial Imagery Crown Width. (DOCX)

S5 Table Field and Airborne Mensuration Data Related to T. nudiflora. (DOCX)

S6 Table Summary Statistics Field and Airborne Mensuration Data. (DOCX)

S7 Table Field and Airborne Mensuration Data Related to Dipterocarpus alatus. (DOCX)

S8 Table Data Summary Field and Airborne Mensuration Data Related to Dipterocarpus alatus. (DOCX)

S9 Table Field and Airborne mensuration Data Related to L. calycuta. (DOCX)

S10 Table Data Summary Field and Airborne mensuration Data Related to L calycuta. (DOCX)

S11 Table Comparison of Producer and User Accuracies for 4 Band ML Classification. (DOCX)
