# Correction: Mapping and Characterizing Selected Canopy Tree Species at the Angkor World Heritage Site in Cambodia Using Aerial Data

**DOI:** 10.1371/journal.pone.0154548

**Published:** 2016-04-26

**Authors:** Minerva Singh, Damian Evans, Boun Suy Tan, Chan Samean Nin

Following publication, a number of errors have been identified in the data analysis and the authors wish to apologise for any inconvenience caused by these errors and to provide corrected data.

There are some errors in the values reported in Table 1. Please see the corrected Table 1 here, with amendments marked in bold, underlined font.

**Table 1:** Field-measured and Light Detection and Ranging (LiDAR)-predicted Mensuration Variables for Some Tree Species.

Tree Species	Tree Height (*m*)			Crown Width (*m*)		
	OBIA-Predicted	Field-Measured	R^a^	OBIA-Predicted	Field-Measured	R^a^
***Dipterocarpus alatus***	35.9**4**±1.39	34.29±1.57	0.416	23.**6**3±1.3**9**	20.**97**±1.2**1**	0.777
***Tetrameles nudiflora***	25.14±2.32	26.**5**±1.9**1**	0.717	13.29±1.2**3**	13.**04** ±1.1**5**	0.754
***Lagerstroemia calyculata***	27.07±1.**60**	31.8±1.66	0.743	14.08±1.**40**	17.63±1.68	0.685

^a^Spearman’s rank coefficient of correlation.

Fig 8 was plotted with a subset of data (using a data set that did not include species-specific data, provided as Tables S1 and S3 of the original article). A new version of Fig 8 plotted using the complete data set is provided here. The complete data set can be found in Tables S5, S7 and S9 of the original article, total n = 113. For both of the graphs in Fig 8 there are new (but still positive) Spearman’s correlation values (0.630 for the tree heights and 0.779 for the crown widths). Please see the complete, corrected Fig 8 here.

**Fig 8:**
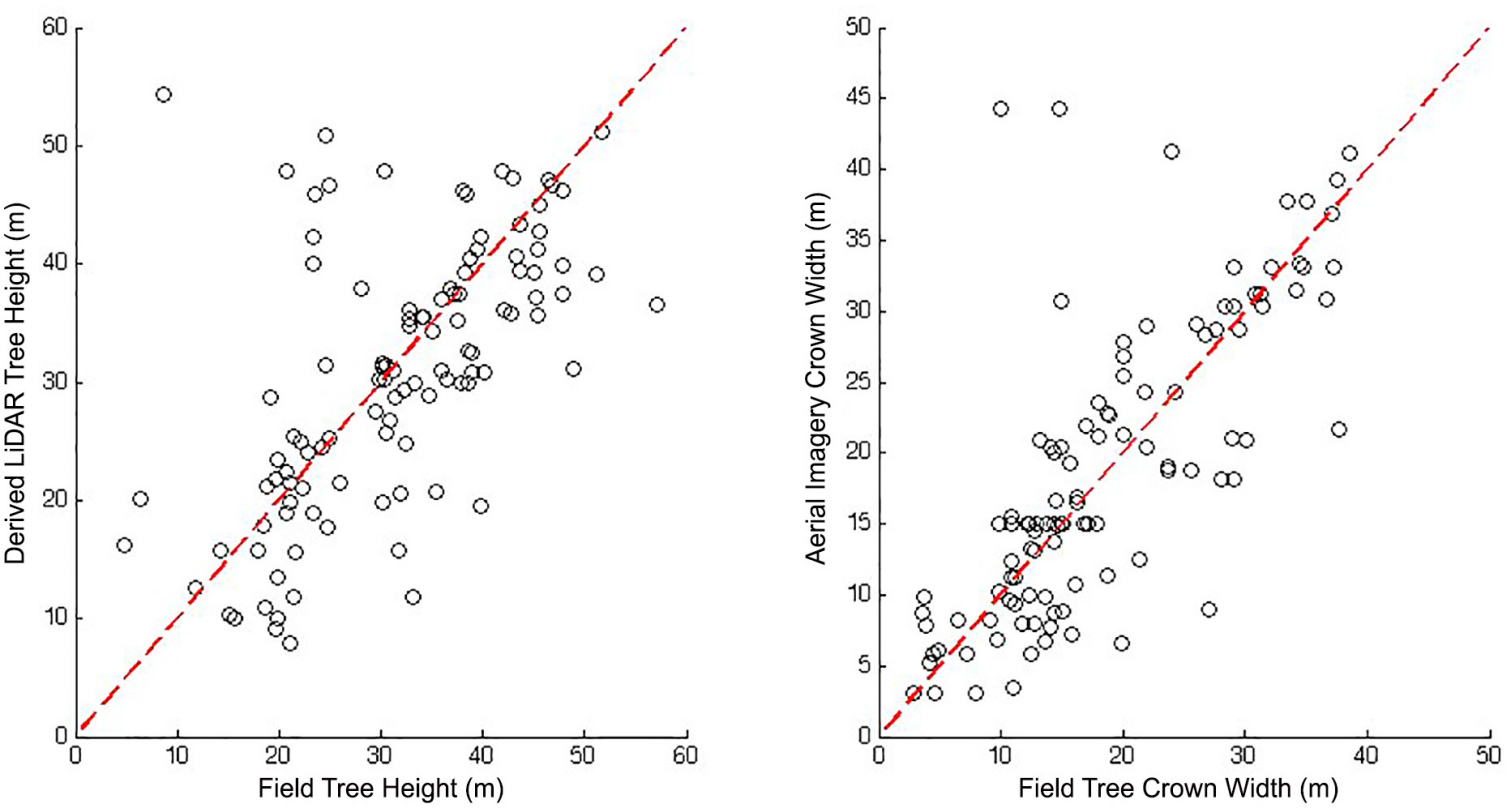
Comparison of Field-measured and Object-based Image Analysis (OBIA)-predicted Crown Widths and Tree Heights sampled for 113 trees. Data sets can be extracted from Tables S5, S7 and S9.

There was an error in the formula used to calculate the RMSE values. The correct values are 9.97m for the height and 6.86m for the crown width.

As a result of these changes, the following revisions are required to the RMSE and correlation values given in the text:

**Abstract**: "Crown width and tree height values that were extracted using multiresolution segmentation showed a high level of congruence with field-measured values of the trees (Spearman’s rho 0.779 and 0.630, respectively)."

**Results**: Forest mensuration variables from the aerial data: "The Spearman’s rank correlation coefficient (rho) between field-measured and LiDAR-derived tree heights was 0.630, whereas rho between field-measured and aerial imagery-derived crown widths was 0.779. A 1:1 line (shown in red) was also fitted, and it can be seen that the predicted values for tree crowns from multiresolution segmentation coincide strongly with the field-measured values. The root-mean-square error (RMSE) for field-measured tree heights and LiDAR heights was 9.97 m, whereas that for crown widths was 6.86 m."

**Discussion**: Comparing multiresolution and watershed segmentation methods: "The field-measured and multiresolution segmentation-extracted crown widths have a strong association with each other (rho = 0.779)."

Use of aerial data for studying forest structure variables: "The LiDAR-derived tree height data have a moderately strong correlation with field-measured heights (rho = 0.630)."

"The RMSE value of 9.97 m and strength of association between field- and LiDAR-derived values in this study is consistent with RMSE values between field-measured and LiDAR tree height data from other tropical ecosystems [86,91], and the LiDAR-derived tree height values are within the range of ground tree height values observed in similar ecosystems [51]."

The authors provide the following discussion of these changes:

The new RMSE values, and the revised Fig 8, do not reverse the conclusions of the article. There is a correlation both with and without species information indicating significant association and correspondence between these LiDAR and field measures, with only the magnitudes of RMSE changed as a result of these corrections.

## References

[1] Singh M, Evans D, Tan BS, Nin CS (2015) Mapping and Characterizing Selected Canopy Tree Species at the Angkor World Heritage Site in Cambodia Using Aerial Data. PLoS ONE 10(4): e0121558 doi: 10.1371/journal.pone.0121558 25902148 10.1371/journal.pone.0121558 PMC4406680

