# Supplementary material for: Mapping and Characterizing Selected Canopy Tree Species at the Angkor World Heritage Site in Cambodia Using Aerial Data
Source: PLoS One. 2015 Apr 22;10(4):e0121558. doi: 10.1371/journal.pone.0121558 (PMC4406680; doi:10.1371/journal.pone.0121558)

**S1 Fig. QQ Plots of Tree Height Residuals- Thick Tail Distributions**

**(Indicative of Non-normal Distribution of Errors)**


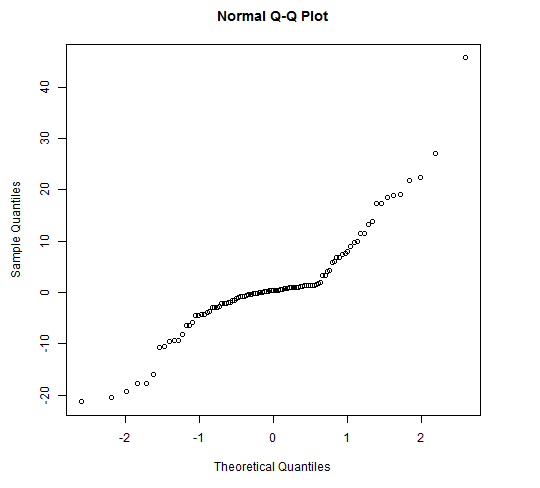

Supplement: S1 Fig — (DOCX) [file pone.0121558.s001.docx]
