# Supplementary figures and images for: Mapping and Characterizing Selected Canopy Tree Species at the Angkor World Heritage Site in Cambodia Using Aerial Data
Source: PLoS One. 2015 Apr 22;10(4):e0121558. doi: 10.1371/journal.pone.0121558 (PMC4406680; doi:10.1371/journal.pone.0121558)

**S3 Fig. QQ Plots for All Three Species**


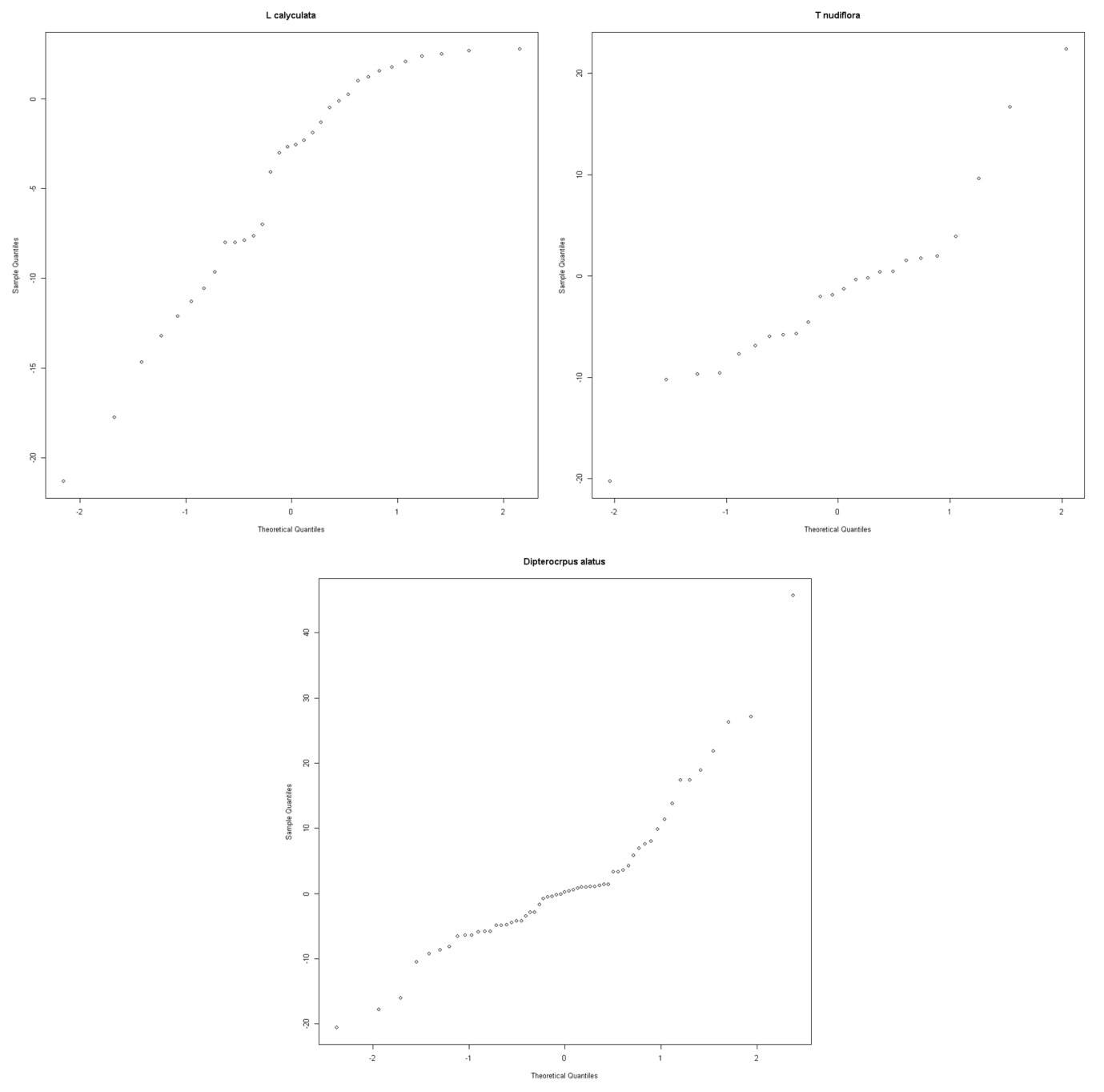

Supplement: S3 Fig — (DOCX) [file pone.0121558.s003.docx]
