# Supplementary material for: Mapping and Characterizing Selected Canopy Tree Species at the Angkor World Heritage Site in Cambodia Using Aerial Data
Source: PLoS One. 2015 Apr 22;10(4):e0121558. doi: 10.1371/journal.pone.0121558 (PMC4406680; doi:10.1371/journal.pone.0121558)
Supplement: S4 Fig — (DOCX) [file pone.0121558.s004.docx]

**S4 Fig. Comparison of Field-measured and Object-based Image Analysis (OBIA)-Predicted Crown Widths and Tree Heights**

**
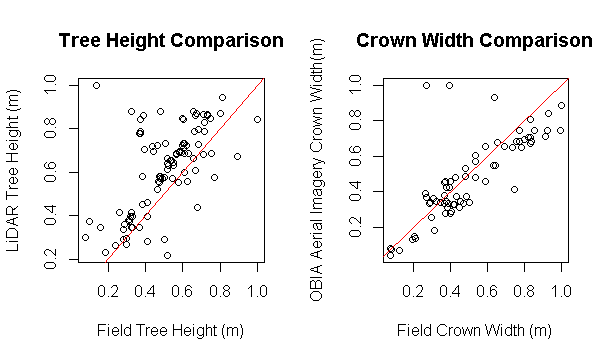
**

This graph is the same as S3 Fig of the manuscript. Only difference is that here XY axis of both the graphs have been normalized from 0 to 1. The red line is the 1:1 line.
