# Supplementary material for: Mapping and Characterizing Selected Canopy Tree Species at the Angkor World Heritage Site in Cambodia Using Aerial Data
Source: PLoS One. 2015 Apr 22;10(4):e0121558. doi: 10.1371/journal.pone.0121558 (PMC4406680; doi:10.1371/journal.pone.0121558)
Supplement: S5 Fig — (DOCX) [file pone.0121558.s005.docx]

**S5 Fig. Spectral Separability of the Different Tree Species Under Consideration**


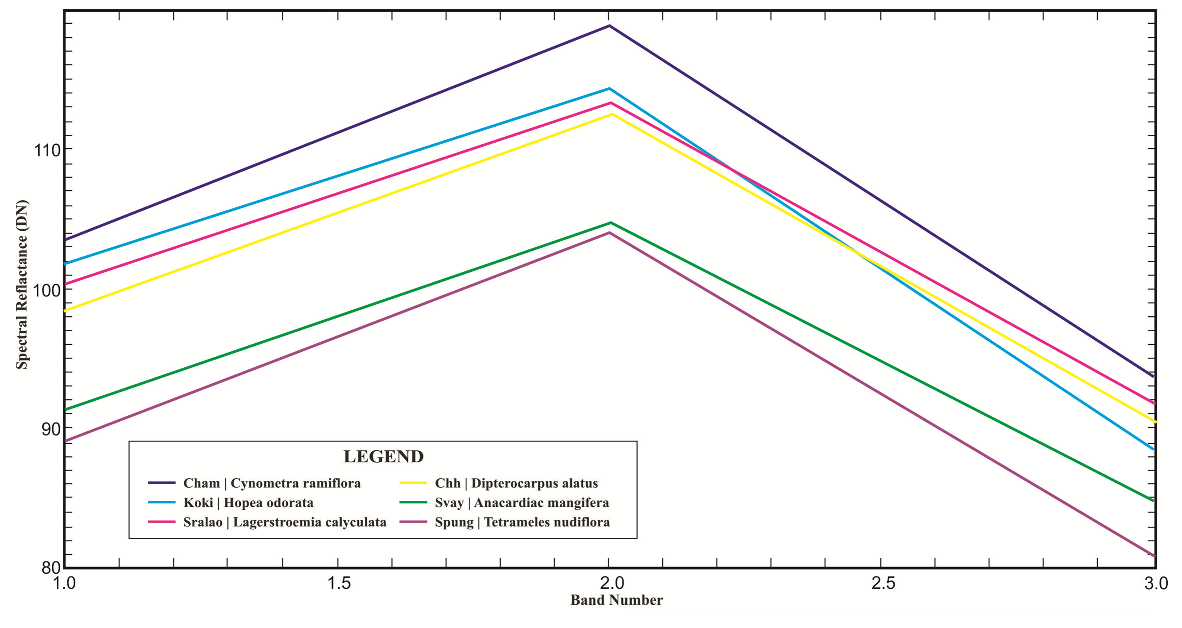


The spectral separability of the different tree species was plotted using the ENVI Image Processing software (<http://www.exelisvis.com/docs/RegionOfInterestTool.html>). Some species, such as Hopea odorata and Dipterocarpus alatus have high separability. Others such as Dipterocarpus alatus, L calycuta and A mangifera have low separability and overlaps. This makes their identification and classification using spectral data alone challenging.
