# Supplementary material for: Mapping and Characterizing Selected Canopy Tree Species at the Angkor World Heritage Site in Cambodia Using Aerial Data
Source: PLoS One. 2015 Apr 22;10(4):e0121558. doi: 10.1371/journal.pone.0121558 (PMC4406680; doi:10.1371/journal.pone.0121558)
Supplement: S6 Fig — (DOCX) [file pone.0121558.s006.docx]

**S6 Fig. Maximum Likelihood Classification of Spectral and Textural Bands for Tree Species Classification (4 Band Imagery)**


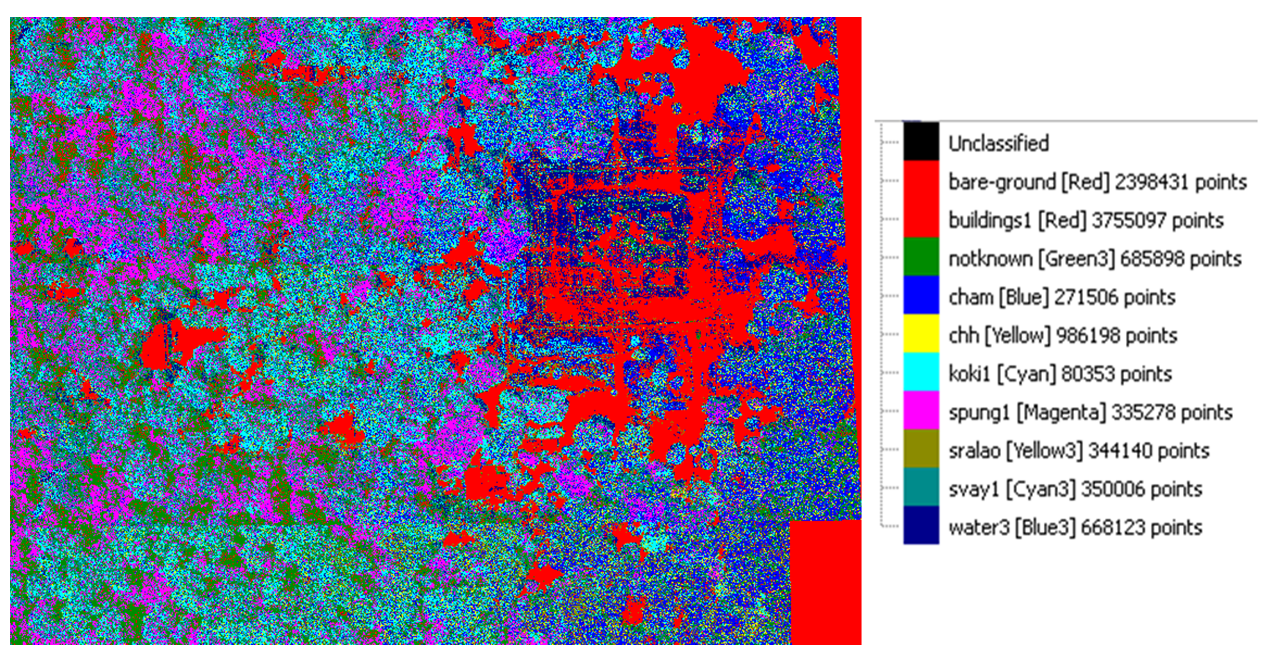


Overall accuracy = 73.8%

Kappa Coefficient = 62.3%
