# Supplementary material for: Mapping and Characterizing Selected Canopy Tree Species at the Angkor World Heritage Site in Cambodia Using Aerial Data
Source: PLoS One. 2015 Apr 22;10(4):e0121558. doi: 10.1371/journal.pone.0121558 (PMC4406680; doi:10.1371/journal.pone.0121558)
Supplement: S7 Fig — (DOCX) [file pone.0121558.s007.docx]

**S7 Fig. Distribution of Crown Widths- of Field Measured Data, eCognition (Multiresolution) Extracted and Watershed Segmentation Extracted**

**
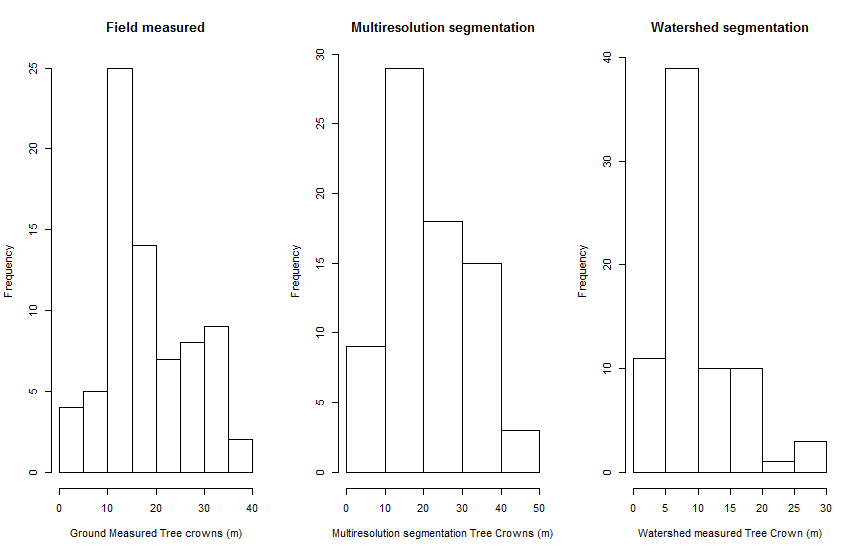
**

Two sided Kolmogorov-Smirnov test was carried out to compare the distributions of field measured crown widths with multi-resolution segmentation derived crown widths. No statistically significant difference exists between their distributions. However distributions of field measured crown widths were found to be significantly different from watershed segmentation derived crown widths.
