# Supplementary material for: Mapping and Characterizing Selected Canopy Tree Species at the Angkor World Heritage Site in Cambodia Using Aerial Data
Source: PLoS One. 2015 Apr 22;10(4):e0121558. doi: 10.1371/journal.pone.0121558 (PMC4406680; doi:10.1371/journal.pone.0121558)
Supplement: S8 Fig — (DOCX) [file pone.0121558.s008.docx]

**LiDAR Data Processing**

**S8 Fig. LiDAR Point Cloud Processing for Deriving CHM and Related Products**

The LiDAR point cloud derived products- DTM, DSM and CHM all had a resolution of 0.5m. FUSION/LDV is a freeware software that provides an intuitive and easy workflow for processing LiDAR point clouds [1]. It has been used for tropical forestry studies for processing LiDAR point clouds and deriving individual and plot scale metrics [2,3]. FUSION/LDV has a utility-“GroundFilter” which uses the ground filtering algorithm. This algorithm identifies the returns that lie close to the ground or the bare earth points. With LiDAR data that have density >4 points/sq km., this algorithm can remove non-ground return effectively. A further algorithm, “Grid Surface Create” is implemented on the bare-ground points to create a gridded surface model or the DTM. The DTM thus created is considered to be adequate for facilitating the calculation of vegetation heights. Please see the following workflow for different processing steps undertaken for generating a CHM from LiDAR point clouds in FUSION/LDV.


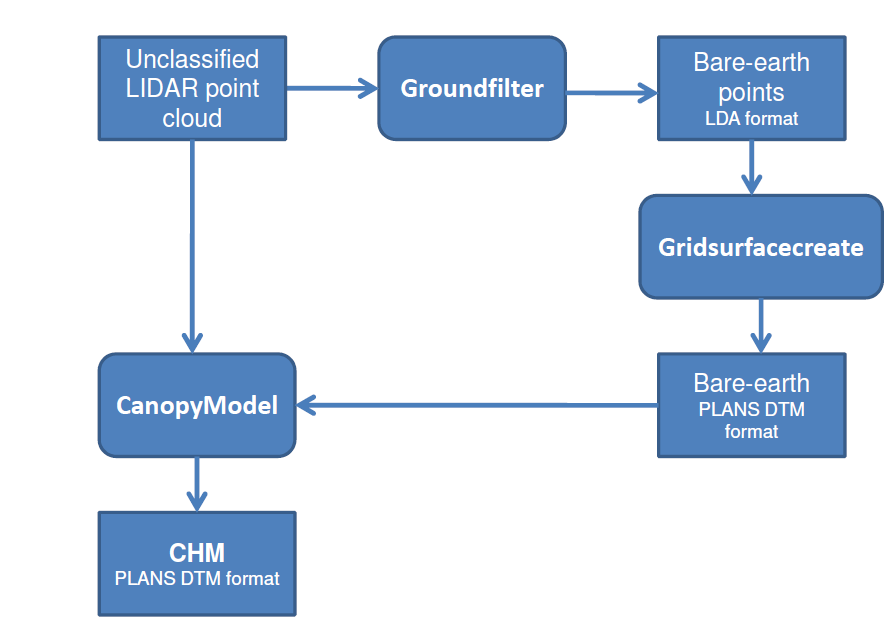


Creation of a ground surface model or DTM involves using the average of elevations from each off the grid cells to produce the gridded/rasterized DTM model. Hence an interpolation step is indeed needed to project the 3D LiDAR point cloud onto a 2D rasterized surface (in this case DTM which in turn is used as an input to generate a CHM). Smoothing is carried out to remove any remainder vegetation (such as shrubs etc) which maybe left on the surface model. However smoothing of the DTM is carried out well after the classification of LiDAR points into bare-earth points and non-ground points and is entirely optional. Hence creation of the gridded raster and its smoothing do not influence the classification of LiDAR points (which as is shown in the flowchart is the primary starting point). Further FUSION/LDV has been designed with forestry applications in mind and can carry out accurate point classifications for LiDAR point clouds with density > 4 sq km.

**References**

1. McGaughey RJ. FUSION/LDV: Software for LIDAR Data Analysis and Visualization: United States Department of Agriculture, Forest Service, Pacific Northwest Research Station; 2010. pp. 154. Available: http://forsys.cfr.washington.edu/fusionlatest.html. Accessed November 2014.

2. Andersen HE, Reutebuch SE, McGaughey RJ, d’Oliveira MVN, Keller M. Monitoring selective logging in western Amazonia with repeat lidar flights. Remote Sens Environ. In press. Available:http://ainfo.cnptia.embrapa.br/digital/bitstream/item/92875/1/Monitoring-selective-logging-in-western-Amazonia-with-repeat-lidar-flights.pdf

3. Silva B, Bendix, J. Remote sensing of vegetation in a tropical mountain ecosystem: individual tree-crown detection. Proc. SPIE 8893, Earth Resources and Environmental Remote Sensing/GIS Applications IV, 88930B (October 24, 2013); doi:10.1117/12.2029912
