# Supplementary material for: Mapping and Characterizing Selected Canopy Tree Species at the Angkor World Heritage Site in Cambodia Using Aerial Data
Source: PLoS One. 2015 Apr 22;10(4):e0121558. doi: 10.1371/journal.pone.0121558 (PMC4406680; doi:10.1371/journal.pone.0121558)
Supplement: S9 Fig — (DOCX) [file pone.0121558.s009.docx]

**S9 Fig. Derive Tree Heights from LiDAR CHM Using Multiresolution Segmentation**

A Crown Height Model (CHM) is calculated by subtracting the height value of the DTM (Digital terrain model) at each pixel from the height value of the DSM (Digital surface model), so that tree heights can be taken directly from the CHM. Each pixel of CHM represents the canopy height above the ground/topography. The trees height extraction might not be redistricted to follow tree crown delineation because each tree may have more than one maximum intensity values. Multi-resolution segmentation splits the image into small polygons/segments on the basis of color variation or intensity from white to dark pixels. The maximum intensity pixels remain in the center of each polygon/segment and can be extracted using extract value to point from each polygon/segment centroid.


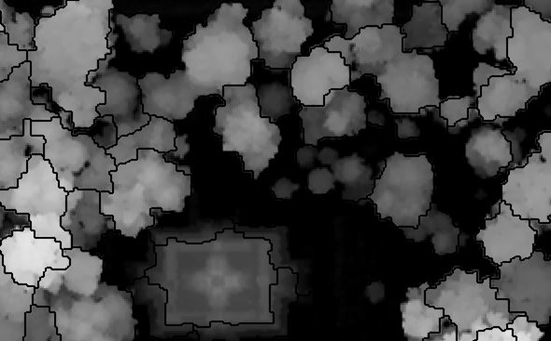


Segmentation of CHM data using Multi-resolution segmentation in ECognition

Multi-resolution segmentation was done on LiDAR CHM data using eCognition software. This segmentation method was developed which encounter the spectral and textural properties of the objects to be detected. This segmentation divides the images into small portions based on certain criteria (shape, compactness and layer weights). Then find the tree crown height from the multi-resolution segmented CHM data having maximum brightness. The segmented CHM trees polygon centers were generated and by using extract value to point extract the maximum intensity from CHM at each polygon.
