# Supplementary material for: Mapping and Characterizing Selected Canopy Tree Species at the Angkor World Heritage Site in Cambodia Using Aerial Data
Source: PLoS One. 2015 Apr 22;10(4):e0121558. doi: 10.1371/journal.pone.0121558 (PMC4406680; doi:10.1371/journal.pone.0121558)
Supplement: S10 Fig — (DOCX) [file pone.0121558.s010.docx]

**Multiresolution Segmentation of VHR Aerial Imagery Using ECognition**

**S10 Fig. Segmented Tree Crowns from Aerial Imagery**


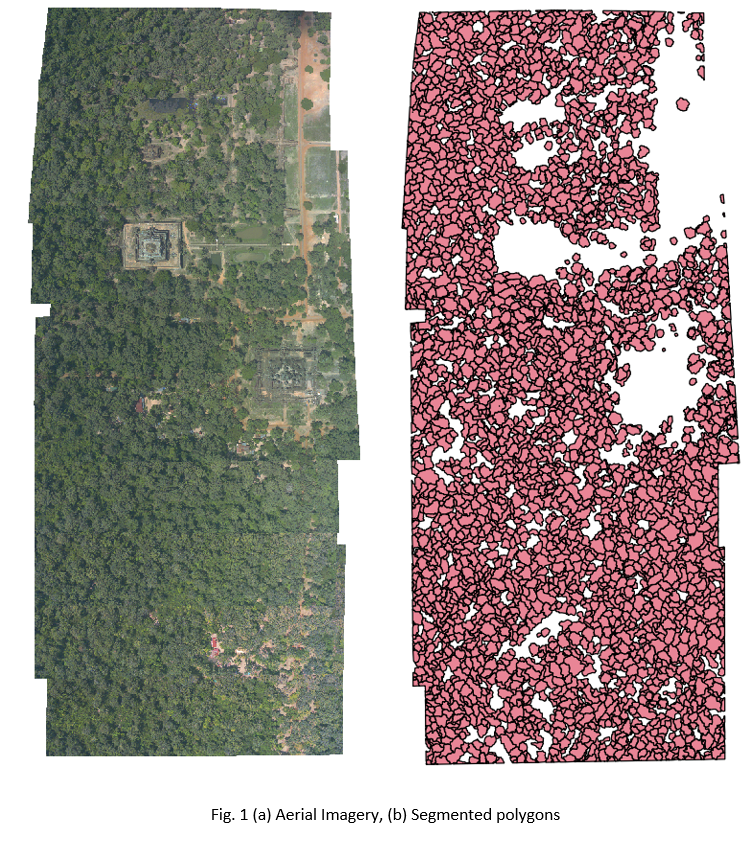


The left image is the actual image and the right one are the tree crowns obtained by implementing multiresolution segmentation on the aerial imagery.
