# Supplementary material for: Mapping and Characterizing Selected Canopy Tree Species at the Angkor World Heritage Site in Cambodia Using Aerial Data
Source: PLoS One. 2015 Apr 22;10(4):e0121558. doi: 10.1371/journal.pone.0121558 (PMC4406680; doi:10.1371/journal.pone.0121558)
Supplement: S11 Fig — (DOCX) [file pone.0121558.s011.docx]

**S11 Fig. Evaluating Segmentation Accuracy Using Goodness-of-Fit**

One of the most commonly adopted methods for evaluating the performance of automatic segmentation (in this case the OBIA based segmentation) is to manually delineate individual tree crowns in the aerial imagery. It is recommended the selection of the tree crowns to be delineated (henceforth to be referred as reference polygons) be carried out randomly. The aerial imagery we are using is very high resolution (8cm), was collected in 2013 and no tree removal has been carried out in the area under consideration. Hence the manually delineated polygons are good representation of the spatial location, size and shape of field tree crowns. Therefore manually delineated tree crowns can be used as proxies for the actual field tree crowns ([Marangoz2014](http://www.academia.edu/2961406/Evaluation_of_Information_Content_and_Feature_Extraction_Capability_of_Very_High_Resolution_QuickBird_Pan-Sharpened_Image)). The aim of the goodness of fit evaluation was to compare the eCognition segmented tree crown polygons with the manually delineated tree crown polygons to see how well the former had approximated the latter/ evaluate how "close" are the algorithm segmented tree crowns to these reference polygons. [64]. Closeness Index (D) estimates the “goodness of polygon matching” between the reference and segmented tree crown polygons [32,65] which gives a measure of accuracy of segmentation.. D value was calculated as shown below (Clinton 2010):

$$D(i, j)=\sqrt{\left( OverSegementation(i,j) \right)^{2}+\left( UnderSegementation(i,j) \right)^{2}}$$

Where,

i = reference polygon id

j = segment polygon id

OverSegmentation (i, j) = $1- \frac{A_{intersection}(i,j)}{A_{reference}(i)}$

UnderSegmentation (i, j) = $1- \frac{A_{intersection}(i,j)}{A_{segment}(j)}$

A_intersect_ (i, j) = Common area between segment – j polygon and corresponding reference polygon – i

A_reference_ (i) = Area of reference polygon – i

A_segment_ (j) = Area of reference polygon – j

The D measure accounts for the over-segmentation and under-segmentation that may have occurred during automated delineation. Under-segmentation means that a segment (in this case, a tree crown segmented from aerial imagery) contains a significant crown part of more than one tree. Over-segmentation refers to more than one segment being associated with a ground tree. Both of these situations are commonly encountered for tree crown segmentation [66]. In addition, by overlaying segmented polygons on reference polygons their areas of overlap are compared which let us evaluate the accuracy of spatial location of segmented tree crowns, their topology and geometric shape [64]. The spatial accuracy is evaluated as closer the manually delineated polygons and eCognition polygons are to each other in terms of their overlap/good ness of fit, more accurate is the spatial location of the eCognition polygon to the manually digitized polygon. As mentioned before, tree crowns manually delineated on VHR imagery act as a good proxy for spatial location of field tree crowns. Again, the goodness of fit approach, is a geometric goodness of fit. The workflow accounts for the geometry of both the manually delineated and eCognition derived polygons, and D value lets us account for both spatial and geometric overlap. A detailed analysis of crown shapes and geometry is beyond the scope of the current research.

The measure D is computed for every pair of reference and segment polygons which have a significant amount of overlap. To find the overall measure D of segmentation accuracy, an average D can be computed for all the segment polygons having significant overlap with the reference polygons. Alternatively, average oversegmentation and undersegmentation can be computed and used to calculate an overall D as per the same formula as above. The average may be weighted or non-weighted. The closer the value of D is to zero, more accurate is the segmentation [67]. Few examples of close matches observed between reference and segmented polygons are shown in S11 Fig.

| 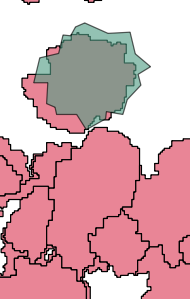 | 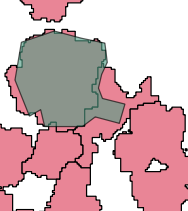 | 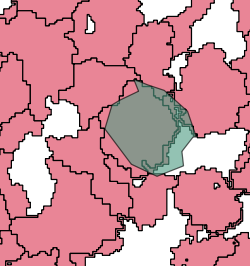 |
| --- | --- | --- |
| 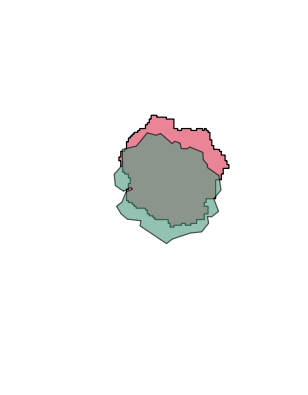 | 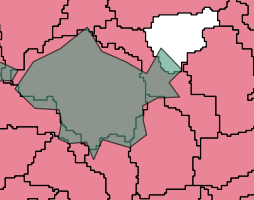 | 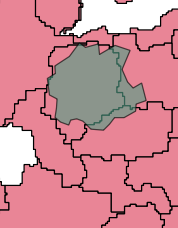 |

Examples of segments and reference polygons with close matches. Reference polygons in green colour and segmented polygons in pink colour

The D measure does not just account for oversegmentation and undersegmentation. Areas of both reference and eCognition polygons are accounted for in this approach which in turn is a valid proxy for tree crown geometry. A detailed study of tree crown geometry is beyond the scope of this research. The spatial accuracy is also accounted for as closer the manually delineated polygons and eCognition polygons are to each other in terms of their overlap/good ness of fit, more accurate is the spatial location of the eCognition polygon to the manually digitized polygon. As mentioned before, tree crowns manually delineated on VHR imagery act as a good proxy for spatial location of field tree crowns.
