# Supplementary material for: Mapping and Characterizing Selected Canopy Tree Species at the Angkor World Heritage Site in Cambodia Using Aerial Data
Source: PLoS One. 2015 Apr 22;10(4):e0121558. doi: 10.1371/journal.pone.0121558 (PMC4406680; doi:10.1371/journal.pone.0121558)
Supplement: S1 Table — (DOCX) [file pone.0121558.s012.docx]

**S1 Table. Field Measured DBH, Tree Height and Corresponding LiDAR Height (These Data Were Collected sans Species Information)**

DBHcm- Diameter at breast height measured in cm using the DBH tape

TreeHt- Field measured tree height in metres

LidHt- LiDAR CHM based tree heights in metres

| **DBHcm** | **TreeHt** | **LidHt** |
| --- | --- | --- |
| 140 | 32.90000 | 33.30743 |
| 80 | 19.90000 | 20.81549 |
| 85 | 20.25743 | 21.61001 |
| 155 | 38.50985 | 38.54280 |
| 147 | 38.48474 | 39.81730 |
| 135 | 29.50000 | 28.27269 |
| 137 | 30.60000 | 30.97123 |
| 138 | 31.20000 | 30.97123 |
| 120 | 34.70000 | 31.00092 |
| 178 | 45.45252 | 47.09264 |
| 180 | 46.50000 | 47.09264 |
| 75 | 11.74000 | 12.54971 |
| 100 | 24.30000 | 24.56786 |
| 110 | 38.90000 | 37.28857 |
| 120 | 51.67088 | 51.20000 |
| 90 | 47.80000 | 46.13422 |
| 90 | 38.12680 | 46.13422 |
| 102 | 30.20000 | 31.60445 |
| 98 | 35.90000 | 36.96104 |
| 118 | 36.90000 | 37.90717 |
| 133 | 42.53875 | 33.15879 |
| 173 | 21.10000 | 21.5256 |
| 96 | 30.30000 | 30.15381 |
| 80 | 18.29742 | 19.47841 |
| 77 | 17.81636 | 18.37678 |
| 80 | 19.90000 | 20.17054 |
| 177 | 29.80000 | 30.20325 |
| 70 | 20.74769 | 18.86175 |
| 82 | 20.97799 | 18.86175 |
| 50 | 42.30000 | 42.75123 |
| 108 | 45.61135 | 42.75123 |
| 70 | 39.60000 | 39.27049 |
| 104 | 19.01000 | 16.02459 |
| 144 | 35.10000 | 34.35300 |
| 130 | 33.35332 | 34.35300 |
| 155 | 32.80000 | 34.72485 |
| 90 | 33.10000 | 33.33406 |
| 61 | 26.10000 | 25.02484 |
| 98 | 40.13462 | 37.30274 |
| 90 | 43.65175 | 39.45111 |
| 108 | 35.90000 | 37.30274 |
| 128 | 31.90000 | 31.17147 |
| 78 | 15.20000 | 14.32113 |
| 60 | 18.82392 | 14.55502 |
| 85 | 25.13075 | 38.30637 |
| 130 | 19.68215 | 20.40372 |
| 170 | 57.10000 | 36.57665 |
| 160 | 45.30495 | 37.11944 |
| 108 | 30.44752 | 31.45154 |
| 82 | 24.55195 | 31.45154 |
| 110 | 32.80159 | 36.12846 |
| 140 | 42.01493 | 36.12846 |
| 105 | 30.44752 | 31.45154 |
| 70 | 24.55195 | 31.45154 |
| 109 | 32.80159 | 36.12846 |
| 60 | 20.67715 | 47.80177 |
| 61 | 23.33525 | 42.28645 |
| 66 | 27.65909 | 39.14417 |
| 111 | 35.17540 | 35.14881 |
| 98 | 30.41904 | 47.80177 |
| 90 | 30.40757 | 47.80177 |
| 135 | 41.97663 | 47.80177 |
| 128 | 38.93982 | 32.41837 |
| 130 | 37.75561 | 37.52626 |
| 140 | 37.11437 | 37.52626 |
| 142 | 47.88793 | 37.40031 |
| 29 | 26.03208 | 21.5256 |
| 62 | 26.04262 | 15.26786 |
| 128 | 48.90567 | 31.17147 |
| 180 | 43.65844 | 43.26871 |
| 120 | 16.48224 | 22.47761 |
| 170 | 36.55287 | 30.15381 |
| 100 | 20.70000 | 22.47761 |
| 80 | 6.331315 | 20.17054 |
| 177 | 39.81379 | 30.20325 |
| 70 | 20.74769 | 18.86175 |
| 82 | 20.97799 | 18.86175 |
| 48 | 9.989566 | 18.86175 |
| 93 | 23.41943 | 18.86175 |
| 45 | 4.877675 | 16.26053 |
| 48 | 43.22045 | 23.8334 |
| 50 | 23.68973 | 42.75123 |
| 108 | 45.61135 | 42.75123 |
| 70 | 29.61299 | 39.27049 |
| 100 | 38.24495 | 39.27049 |
| 125 | 31.77995 | 15.78571 |
| 90 | 17.84448 | 15.78571 |
| 132 | 38.33695 | 45.89752 |
| 90 | 23.55852 | 45.92529 |
| 5 | 23.75936 | 42.30942 |
| 100 | 63.73473 | 45.92529 |
| 210 | 8.628319 | 54.32086 |
| 100 | 33.10214 | 11.80621 |
| 125 | 51.25005 | 47.25323 |
| 117 | 43.00338 | 47.25323 |
| 106 | 42.6881 | 46.69748 |
| 100 | 46.78087 | 46.69748 |
| 94 | 24.85848 | 46.69748 |
| 102 | 32.34565 | 39.76272 |
| 87 | 38.90000 | 39.76272 |
| 95 | 34.2178 | 35.47807 |
| 108 | 34.10000 | 35.47807 |
| 103 | 45.60000 | 45.03828 |
| 118 | 28.01172 | 37.90717 |
| 133 | 42.53875 | 33.15879 |
