# Supplementary material for: Mapping and Characterizing Selected Canopy Tree Species at the Angkor World Heritage Site in Cambodia Using Aerial Data
Source: PLoS One. 2015 Apr 22;10(4):e0121558. doi: 10.1371/journal.pone.0121558 (PMC4406680; doi:10.1371/journal.pone.0121558)
Supplement: S2 Table — (DOCX) [file pone.0121558.s013.docx]

**S2 Table. Data Summary of Field Measured DBH, Tree Height and Corresponding LiDAR Height**

| **DBH cm** | | **Tree Ht** | | **Lid Ht** | |
| --- | --- | --- | --- | --- | --- |
| Min. | 5.0 | Min. | 4.878 | Min. | 11.81 |
| 1^st^ Qu. | 82.0 | 1^st^ Qu. | 23.690 | 1^st^ Qu. | 23.83 |
| Median | 102.0 | Median | 32.800 | Median | 35.15 |
| Mean | 106.4 | Mean | 32.036 | Mean | 33.29 |
| 3^rd^ Qu. | 130.0 | 3^rd^ Qu. | 39.600 | 3^rd^ Qu. | 39.82 |
| Max. | 210.0 | Max. | 63.735 | Max. | 54.32 |
