# Supplementary material for: Mapping and Characterizing Selected Canopy Tree Species at the Angkor World Heritage Site in Cambodia Using Aerial Data
Source: PLoS One. 2015 Apr 22;10(4):e0121558. doi: 10.1371/journal.pone.0121558 (PMC4406680; doi:10.1371/journal.pone.0121558)
Supplement: S3 Table — (DOCX) [file pone.0121558.s014.docx]

**S3 Table. Field Measured DBH, Field Measured Crown Width, OBIA Extracted Aerial Imagery Crown Width, Watershed Segmented Aerial Imagery Crown Width (These Data too Were Collected sans Species Information)**

| **DBHcm** | **FieldCrown** | **AerialCrown** | **WaterCrwn** |
| --- | --- | --- | --- |
| 140 | 18 | 21.53299 | 11.28379 |
| 80 | 20 | 26.76621 | 15.1388 |
| 85 | 24.1 | 24.20252 | 15.1388 |
| 155 | 26 | 29.11361 | 3.568248 |
| 147 | 28 | 18.1813 | 3.568248 |
| 135 | 30 | 30.76102 | 15.55363 |
| 137 | 16 | 21.34268 | 15.55363 |
| 138 | 20 | 21.34268 | 15.55363 |
| 120 | 22 | 20.3981 | 24.97774 |
| 178 | 27.6 | 28.65206 | 25.731 |
| 180 | 29.5 | 28.65206 | 25.731 |
| 75 | 14 | 12.33343 | 3.568248 |
| 100 | 11.2 | 11.26621 | 3.6 |
| 110 | 17 | 15.25212 | 15.55363 |
| 120 | 16.2 | 16.53611 | 11.28379 |
| 90 | 14.8 | 44.22703 | 16.35177 |
| 90 | 10 | 44.22703 | 8.740387 |
| 102 | 18 | 23.5 | 25.23133 |
| 98 | 14.4 | 13.8 | 5.046265 |
| 118 | 22 | 29.0045 | 5.5 |
| 133 | 31.2 | 35.77653 | 6.2 |
| 173 | 11 | 15.03315 | 8.740387 |
| 96 | 4.6 | 3.070212 | 6.2 |
| 80 | 15.8 | 14.61736 | 6.180387 |
| 77 | 15.5 | 14.61736 | 5.046265 |
| 80 | 11.8 | 8.009552 | 5.046265 |
| 177 | 13.7 | 20 | 6.180387 |
| 70 | 17.7 | 14.9 | 6.2 |
| 82 | 14.1 | 15.2 | 6.3 |
| 50 | 11.5 | 16.1 | 6.180387 |
| 108 | 9.9 | 17.2 | 8.740387 |
| 70 | 12.1 | 15.2 | 8.5 |
| 104 | 18.2 | 16.53611 | 8.9 |
| 144 | 14.9 | 12.3 | 9.12 |
| 130 | 12.9 | 15.02 | 8.7 |
| 155 | 18.9 | 15.1 | 8.8 |
| 90 | 10.1 | 16.2 | 8.67 |
| 61 | 14.8 | 18.9 | 9.1 |
| 98 | 10.8 | 14.9 | 11.28379 |
| 90 | 15.1 | 12.8 | 6.2 |
| 108 | 12.9 | 15.03315 | 5.9 |
| 128 | 13.8 | 14.7 | 9 |
| 78 | 7.2 | 5.803778 | 5.046265 |
| 60 | 31.2 | 29.84757 | 19.4 |
| 85 | 7.7 | 6.6 | 11.28379 |
| 130 | 23.7 | 24.16581 | 9.1 |
| 170 | 20.1 | 25.37711 | 12.8655 |
| 160 | 24 | 41.32989 | 6.180387 |
| 108 | 15 | 20.37788 | 16.35177 |
| 82 | 14 | 20.37788 | 11.28379 |
| 110 | 28.4 | 30.31376 | 12.8655 |
| 140 | 31.4 | 30.31376 | 6.180387 |
| 105 | 15 | 20.37788 | 16.35177 |
| 70 | 14 | 20.37788 | 11.28379 |
| 109 | 29.1 | 30.31376 | 5.046265 |
| 60 | 29.1 | 33.12337 | 7.978846 |
| 61 | 14.6 | 14.9 | 3.568248 |
| 66 | 24.6 | 30.04508 | 4 |
| 111 | 34.9 | 37.15498 | 5.046265 |
| 98 | 32.1 | 33.12337 | 5.046265 |
| 90 | 34.7 | 33.12337 | 3.568248 |
| 135 | 37.3 | 33.12337 | 3.568248 |
| 128 | 34.2 | 31.42834 | 12.36077 |
| 130 | 31.2 | 31.28121 | 5.046265 |
| 140 | 30.84 | 31.28121 | 3.568248 |
| 142 | 37.6 | 39.28496 | 3.568248 |
| 29 | 16.9 | 13.9 | 8.740387 |
| 62 | 14.1 | 18.7 | 8.740387 |
| 128 | 13.8 | 16.9 | 8.740387 |
| 180 | 2.9 | 3.4 | 8.740387 |
| 120 | 2.8 | 1.9 | 6.180387 |
| 170 | 7.9 | 6.2 | 6.180387 |
| 100 | 2.7 | 3.5 | 3.5 |
| 80 | 11.8 | 8.009552 | 10.7 |
