# Supplementary material for: Mapping and Characterizing Selected Canopy Tree Species at the Angkor World Heritage Site in Cambodia Using Aerial Data
Source: PLoS One. 2015 Apr 22;10(4):e0121558. doi: 10.1371/journal.pone.0121558 (PMC4406680; doi:10.1371/journal.pone.0121558)
Supplement: S4 Table — (DOCX) [file pone.0121558.s015.docx]

**S4 Table. Data Summary Field Measured DBH, Field Measured Crown Width, OBIA Extracted Aerial Imagery Crown Width, Watershed Segmented Aerial Imagery Crown Width**

| **DBH cm** | | **Field Crown** | | **Aerial Crown** | | **Water Crown** | |
| --- | --- | --- | --- | --- | --- | --- | --- |
| Min. | 29 | Min. | 2.70 | Min. | 1.90 | Min. | 3.500 |
| 1^st^ Qu. | 82 | 1^st^ Qu. | 13.10 | 1^st^ Qu. | 14.90 | 1^st^ Qu. | 5.160 |
| Median | 108 | Median | 15.90 | Median | 19.45 | Median | 8.720 |
| Mean | 110 | Mean | 18.72 | Mean | 20.92 | Mean | 9.378 |
| 3^rd^ Qu. | 135 | 3^rd^ Qu. | 25.65 | 3^rd^ Qu. | 29.66 | 3^rd^ Qu. | 11.284 |
| Max. | 180 | Max. | 37.60 | Max. | 44.23 | Max. | 25.731 |

Here Field Crown- field measured tree crown widths

Aerial crown- OBIA extracted aerial imagery tree crowns

Water Crown: Tree crown widths obtained from watershed segmentation of aerial imagery
