# Supplementary material for: Mapping and Characterizing Selected Canopy Tree Species at the Angkor World Heritage Site in Cambodia Using Aerial Data
Source: PLoS One. 2015 Apr 22;10(4):e0121558. doi: 10.1371/journal.pone.0121558 (PMC4406680; doi:10.1371/journal.pone.0121558)
Supplement: S5 Table — (DOCX) [file pone.0121558.s016.docx]

**S5 Table. Field and Airborne Mensuration Data Related to T. nudiflora**

| **SPECIES** | **Tree_Height** | **CHM_Height** | **CrownDiam** | **CD_aerial** |
| --- | --- | --- | --- | --- |
| spng | 31.2 | 30.97123 | 20 | 21.34268 |
| spng | 21.1 | 21.5256 | 11 | 3.456 |
| spng | 29.8 | 30.20325 | 13.7 | 9.871 |
| spng | 32.8 | 34.72485 | 17.1 | 15.03315 |
| spng | 43.65844 | 43.26871 | 2.9 | 3.070212 |
| spng | 39.81379 | 19.56 | 13.7 | 6.78 |
| spng | 23.41943 | 18.86175 | 18.7 | 11.321 |
| spng | 17.84448 | 15.78571 | 12.9 | 15.03315 |
| spng | 23.55852 | 45.92529 | 17 | 21.9 |
| spng | 42.6881 | 35.78 | 25.6 | 18.765 |
| spng | 45 | 39.2 | 4.1 | 5.2 |
| spng | 21.5 | 25.4 | 3.9 | 7.8 |
| spng | 18.7 | 10.985 | 11.2 | 9.4 |
| spng | 19.1 | 28.7 | 10.9 | 15.453 |
| spng | 38.7 | 40.421 | 17.987 | 21.2 |
| spng | 21.6 | 15.6 | 18.7 | 22.772 |
| spng | 19.8 | 10.1 | 12.8 | 14.5 |
| spng | 21.5 | 11.9 | 13.2 | 20.876 |
| spng | 15.7 | 9.987 | 3.5 | 8.7 |
| spng | 21.1 | 19.8 | 10.7 | 9.6 |
| spng | 20.8 | 18.9 | 14.5 | 16.7 |
| spng | 14.2 | 15.7 | 12.8 | 13.1 |
| spng | 23.4 | 40.1 | 9.9 | 10.2 |
| spng | 30.1 | 19.871 | 16.2 | 16.9 |
