# Supplementary material for: Mapping and Characterizing Selected Canopy Tree Species at the Angkor World Heritage Site in Cambodia Using Aerial Data
Source: PLoS One. 2015 Apr 22;10(4):e0121558. doi: 10.1371/journal.pone.0121558 (PMC4406680; doi:10.1371/journal.pone.0121558)
Supplement: S6 Table — (DOCX) [file pone.0121558.s017.docx]

**S6 Table. Summary Statistics Field and Airborne Mensuration Data**

| **##** | **SPECIES** | | **Tree Ht** | | **CHM Ht** | | **CrownDiam** | | **CD_aerial** | |
| --- | --- | --- | --- | --- | --- | --- | --- | --- | --- | --- |
| ## | chh | 0 | Min | 14.2 | Min. | 9.99 | Min. | 2.9 | Min | 3.07 |
| ## | spng | 24 | 1^st^ Qu. | 20.6 | 1^st^ Qu. | 15.76 | 1^st^ Qu. | 10.8 | 1^st^ Qu. | 9.22 |
| ## | srl | 0 | Median | 22.5 | Median | 20.70 | Median | 13.1 | Median | 13.80 |
| ## |  |  | Mean | 26.6 | Mean | 25.14 | Mean | 13.0 | Mean | 13.29 |
| ## |  |  | 3^rd^ Qu. | 31.6 | 3^rd^ Qu. | 34.99 | 3^rd^ Qu. | 17.0 | 3^rd^ Qu. | 17.37 |
| ## |  |  | Max. | 45.0 | Max. | 45.93 | Max. | 25.6 | Max. | 22.77 |
