# Supplementary material for: Mapping and Characterizing Selected Canopy Tree Species at the Angkor World Heritage Site in Cambodia Using Aerial Data
Source: PLoS One. 2015 Apr 22;10(4):e0121558. doi: 10.1371/journal.pone.0121558 (PMC4406680; doi:10.1371/journal.pone.0121558)
Supplement: S7 Table — (DOCX) [file pone.0121558.s018.docx]

**S7 Table. Field and Airborne Mensuration Data Related to Dipterocarpus alatus**

| **SPECIES** | **Tree_Height** | **CHM_Height** | **CrownDiam** | **CD_aerial** |
| --- | --- | --- | --- | --- |
| chh | 19.9 | 23.5 | 20 | 26.76621 |
| chh | 38.50985 | 32.67 | 26 | 29.11361 |
| chh | 38.48474 | 29.87 | 28 | 18.1813 |
| chh | 34.7 | 28.87 | 22 | 20.3981 |
| chh | 45.45252 | 41.2 | 27.6 | 28.65206 |
| chh | 46.5 | 47.09264 | 29.5 | 28.65206 |
| chh | 11.74 | 12.54971 | 14 | 7.678 |
| chh | 24.3 | 24.56786 | 11.2 | 11.26621 |
| chh | 51.67088 | 51.2 | 16.2 | 16.53611 |
| chh | 47.8 | 46.13422 | 14.8 | 44.22703 |
| chh | 38.1268 | 46.13422 | 10 | 44.22703 |
| chh | 30.2 | 31.60445 | 18 | 23.5 |
| chh | 35.9 | 36.96104 | 14.4 | 13.8 |
| chh | 36.9 | 37.90717 | 22 | 29.0045 |
| chh | 19.9 | 13.467 | 11.8 | 8.009552 |
| chh | 45.61135 | 42.75123 | 9.9 | 15.03315 |
| chh | 35.1 | 34.353 | 14.9 | 15.03315 |
| chh | 33.35332 | 29.879 | 12.9 | 15.03315 |
| chh | 40.13462 | 30.877 | 10.8 | 15.03315 |
| chh | 43.65175 | 39.45111 | 15.1 | 15.03315 |
| chh | 35.9 | 30.986 | 12.9 | 15.03315 |
| chh | 15.2 | 10.32 | 7.2 | 5.803778 |
| chh | 57.1 | 36.57665 | 20.1 | 25.37711 |
| chh | 45.30495 | 37.11944 | 24 | 41.32989 |
| chh | 30.44752 | 25.67 | 15 | 30.77 |
| chh | 24.55195 | 50.87 | 14 | 20.37788 |
| chh | 32.80159 | 36.12846 | 28.4 | 30.31376 |
| chh | 42.01493 | 36.12846 | 31.4 | 30.31376 |
| chh | 30.44752 | 31.45154 | 15 | 20.37788 |
| chh | 24.55195 | 31.45154 | 14 | 20.37788 |
| chh | 32.80159 | 36.12846 | 29.1 | 30.31376 |
| chh | 20.67715 | 47.80177 | 29.1 | 33.12337 |
| chh | 23.33525 | 42.28645 | 14.6 | 14.9 |
| chh | 30.41904 | 47.80177 | 32.1 | 33.12337 |
| chh | 30.40757 | 47.80177 | 34.7 | 33.12337 |
| chh | 41.97663 | 47.80177 | 37.3 | 33.12337 |
| chh | 38.93982 | 32.41837 | 34.2 | 31.42834 |
| chh | 37.75561 | 37.52626 | 31.2 | 31.28121 |
| chh | 37.11437 | 37.52626 | 30.84 | 31.28121 |
| chh | 47.88793 | 37.40031 | 37.6 | 39.28496 |
| chh | 26.03208 | 21.5256 | 16.9 | 15.03315 |
| chh | 36.55287 | 30.15381 | 7.9 | 3.070212 |
| chh | 6.331315 | 20.17054 | 11.8 | 8.009552 |
| chh | 4.877675 | 16.26053 | 14.3 | 15.03315 |
| chh | 45.61135 | 42.75123 | 12.3 | 15.03315 |
| chh | 38.24495 | 39.27049 | 17.9 | 15.03315 |
| chh | 31.77995 | 15.78571 | 12.2 | 15.03315 |
| chh | 38.33695 | 45.89752 | 4.9 | 6.034943 |
| chh | 63.73473 | 45.92529 | 38.5 | 41.19392 |
| chh | 8.628319 | 54.32086 | 34.5 | 33.29834 |
| chh | 43.00338 | 47.25323 | 26.7 | 28.36294 |
| chh | 46.78087 | 46.69748 | 24.3 | 24.33119 |
| chh | 24.85848 | 46.69748 | 21.8 | 24.33119 |
| chh | 34.2178 | 35.47807 | 35 | 37.74536 |
| chh | 34.1 | 35.47807 | 33.4 | 37.74536 |
| chh | 45.6 | 45.03828 | 18.9 | 22.6416 |
| chh | 28.01172 | 37.90717 | 22 | 29.0045 |
