# Supplementary material for: Mapping and Characterizing Selected Canopy Tree Species at the Angkor World Heritage Site in Cambodia Using Aerial Data
Source: PLoS One. 2015 Apr 22;10(4):e0121558. doi: 10.1371/journal.pone.0121558 (PMC4406680; doi:10.1371/journal.pone.0121558)
Supplement: S8 Table — (DOCX) [file pone.0121558.s019.docx]

**S8 Table. Data Summary Field and Airborne Mensuration Data Related to Dipterocarpus alatus**

| **SPECIES** |  | | **Tree Ht** | | **CHM Ht** | | **CrownDiam** | | **CD_aerial** | |
| --- | --- | --- | --- | --- | --- | --- | --- | --- | --- | --- |
| ## | chh | 57 | Min | 4.88 | Min. | 10.3 | Min. | 4.9 | Min | 3.07 |
| ## | spng | 0 | 1^st^ Qu. | 28.01 | 1^st^ Qu. | 30.9 | 1^st^ Qu. | 14.0 | 1^st^ Qu. | 15.03 |
| ## | srl | 0 | Median | 35.90 | Median | 37.0 | Median | 18.9 | Median | 24.33 |
| ## |  |  | Mean | 34.29 | Mean | 35.9 | Mean | 21.0 | Mean | 23.63 |
| ## |  |  | 3^rd^ Qu. | 42.01 | 3^rd^ Qu. | 45.9 | 3^rd^ Qu. | 29.1 | 3^rd^ Qu. | 31.28 |
| ## |  |  | Max. | 63.73 | Max. | 54.3 | Max. | 38.5 | Max. | 44.23 |
