# Supplementary material for: Mapping and Characterizing Selected Canopy Tree Species at the Angkor World Heritage Site in Cambodia Using Aerial Data
Source: PLoS One. 2015 Apr 22;10(4):e0121558. doi: 10.1371/journal.pone.0121558 (PMC4406680; doi:10.1371/journal.pone.0121558)
Supplement: S9 Table — (DOCX) [file pone.0121558.s020.docx]

**S9 Table. Field and Airborne mensuration Data Related to L. calycuta**

| **SPECIES** | **Tree_Height** | **CHM_Height** | **CrownDiam** | **CD_aerial** |
| --- | --- | --- | --- | --- |
| srl | 30.3 | 30.15381 | 4.6 | 3.070212 |
| srl | 31.9 | 20.6 | 13.8 | 15.03315 |
| srl | 19.68215 | 9.12 | 23.7 | 19.012 |
| srl | 48.90567 | 31.17147 | 13.8 | 15.03315 |
| srl | 33.10214 | 11.80621 | 12.5 | 5.78 |
| srl | 51.25005 | 39.123 | 27 | 8.97 |
| srl | 32.34565 | 29.312 | 36.7 | 30.872 |
| srl | 18.4 | 17.9 | 10.878 | 12.334 |
| srl | 21.1 | 7.87 | 9.121 | 8.234 |
| srl | 47.775 | 39.87 | 14.324 | 8.765 |
| srl | 35.432 | 20.761 | 19.875 | 6.543 |
| srl | 45.324 | 35.67 | 4.5 | 5.8 |
| srl | 37.876 | 29.873 | 10.8 | 11.3 |
| srl | 24.786 | 17.765 | 9.7 | 6.8 |
| srl | 39.875 | 42.345 | 12.76 | 7.97 |
| srl | 20.786 | 22.334 | 15.112 | 8.9 |
| srl | 32.4 | 24.765 | 3.786 | 9.87 |
| srl | 43.231 | 40.677 | 6.541 | 8.213 |
| srl | 18.876 | 21.232 | 15.871 | 7.233 |
| srl | 30.87 | 26.78 | 28.91 | 20.98 |
| srl | 24.987 | 25.231 | 12.342 | 9.976 |
| srl | 29.431 | 27.546 | 20.11 | 27.87 |
| srl | 22.134 | 24.876 | 23.65 | 18.76 |
| srl | 30.234 | 31.223 | 30.121 | 20.945 |
| srl | 19.654 | 21.723 | 12.43 | 13.21 |
| srl | 32.768 | 35.418 | 37.7 | 21.654 |
| srl | 39.428 | 41.187 | 16.1 | 10.8 |
| srl | 22.312 | 20.98 | 15.7 | 19.3 |
| srl | 31.48 | 28.78 | 21.3 | 12.45 |
| srl | 37.546 | 35.231 | 14.32 | 19.98 |
| srl | 22.9 | 24.1 | 29.1 | 18.2 |
| srl | 38.9 | 30.9 | 37.1 | 36.83142 |
