# Supplementary material for: Mapping and Characterizing Selected Canopy Tree Species at the Angkor World Heritage Site in Cambodia Using Aerial Data
Source: PLoS One. 2015 Apr 22;10(4):e0121558. doi: 10.1371/journal.pone.0121558 (PMC4406680; doi:10.1371/journal.pone.0121558)
Supplement: S10 Table — (DOCX) [file pone.0121558.s021.docx]

**S10 Table. Data Summary Field and Airborne mensuration Data Related to L calycuta**

| **SPECIES** |  | | **Tree Ht** | | **CHM Ht** | | **CrownDiam** | | **CD_aerial** | |
| --- | --- | --- | --- | --- | --- | --- | --- | --- | --- | --- |
| ## | chh | 0 | Min | 18.4 | Min. | 7.87 | Min. | 3.79 | Min | 3.07 |
| ## | spng | 0 | 1^st^ Qu. | 22.8 | 1^st^ Qu. | 21.17 | 1^st^ Qu. | 11.98 | 1^st^ Qu. | 8.23 |
| ## | srl | 32 | Median | 31.7 | Median | 27.16 | Median | 14.72 | Median | 11.82 |
| ## |  |  | Mean | 31.8 | Mean | 27.07 | Mean | 17.63 | Mean | 14.08 |
| ## |  |  | 3^rd^ Qu. | 38.1 | 3^rd^ Qu. | 32.23 | 3^rd^ Qu. | 23.66 | 3^rd^ Qu. | 19.08 |
| ## |  |  | Max. | 51.2 | Max. | 42.34 | Max. | 37.70 | Max. | 36.83 |

Tree Ht: Field measured tree height

CHM Ht: LiDAR CHM tree height

CrownDiam: Field measured crown diameter

CD_aerial: Crown diameter from aerial imagery
