# Supplementary material for: Mapping and Characterizing Selected Canopy Tree Species at the Angkor World Heritage Site in Cambodia Using Aerial Data
Source: PLoS One. 2015 Apr 22;10(4):e0121558. doi: 10.1371/journal.pone.0121558 (PMC4406680; doi:10.1371/journal.pone.0121558)
Supplement: S11 Table — (DOCX) [file pone.0121558.s022.docx]

**S11 Table.** **Comparison of Producer and User Accuracies for 4 Band ML Classification**

|  | **Buildings** | **CHAM** | **CHH** | **KOKI** | **SPUNG** | **SVAY** | **SRL** | **Water** | **Unknown** | **Bareground** |
| --- | --- | --- | --- | --- | --- | --- | --- | --- | --- | --- |
| **Prod. Acc.%** | 88.2 | 44.1 | 8 | 83.4 | 78.9 | 78.3 | 1.8 | 76.1 | 72.4 | 97.9 |
| **User Acc.%** | 6.8 | 17.9 | 20 | 85.4 | 94.4 | 26.3 | 0.7 | 45.6 | 75.2 | 92.5 |

CHAM: *Cynometra ramiflora* CHH: *Dipterocarpus alatus;* KOKI: *Hopea odorata;* SVAY: *Anacardiac mangifera*; SPUNG: *Tetrameles nudiflora;* and SRL: *Lagerstroemia calyculata*
